# Homology of the Fifth Epibranchial and Accessory Elements of the Ceratobranchials among Gnathostomes: Insights from the Development of Ostariophysans

**DOI:** 10.1371/journal.pone.0062389

**Published:** 2013-04-18

**Authors:** Murilo Carvalho, Flávio Alicino Bockmann, Marcelo Rodrigues de Carvalho

**Affiliations:** 1 Laboratório de Ictiologia de Ribeirão Preto (LIRP), Departamento de Biologia, Faculdade de Filosofia, Ciências e Letras de Ribeirão Preto, Universidade de São Paulo, PPG Biologia Comparada, Ribeirão Preto, SP, Brazil; 2 Laboratório de Ictiologia, Departamento de Zoologia, Instituto de Biociências, Universidade de São Paulo, São Paulo, SP, Brazil; Monash University, Australia

## Abstract

Epibranchials are among the main dorsal elements of the gill basket in jawed vertebrates (Gnathostomata). Among extant fishes, chondrichthyans most resemble the putative ancestral condition as all branchial arches possess every serially homologous piece. In osteichthyans, a primitive rod-like epibranchial 5, articulated to ceratobranchial 5, is absent. Instead, epibranchial 5 of many actinopterygians is here identified as an accessory element attached to ceratobranchial 4. Differences in shape and attachment of epibranchial 5 in chondrichthyans and actinopterygians raised suspicions about their homology, prompting us to conduct a detailed study of the morphology and development of the branchial basket of three ostariophysans (*Prochilodus argenteus*, Characiformes; *Lophiosilurus alexandri* and *Pseudoplatystoma corruscans*, Siluriformes). Results were interpreted within a phylogenetic context of major gnathostome lineages. Developmental series strongly suggest that the so-called epibranchial 5 of actinopterygians does not belong to the epal series because it shares the same chondroblastic layer with ceratobranchial 4 and its ontogenetic emergence is considerably late. This neomorphic structure is called *accessory element of ceratobranchial 4*. Its distribution among gnathostomes indicates it is a teleost synapomorphy, occurring homoplastically in Polypteriformes, whereas the loss of the true epibranchial 5 is an osteichthyan synapomorphy. The origin of the accessory element of ceratobranchial 4 appears to have occurred twice in osteichthyans, but it may have a single origin; in this case, the accessory element of ceratobranchial 4 would represent a remnant of a series of elements distally attached to ceratobranchials 1–4, a condition totally or partially retained in basal actinopterygians. Situations wherein a structure is lost while a similar neomorphic element is present may lead to erroneous homology assessments; these can be avoided by detailed morphological and ontogenetic investigations interpreted in the light of well-supported phylogenetic hypotheses.

## Introduction

Jawed vertebrates, or gnathostomes, are a monophyletic group composed of two extant lineages: Osteichthyes (bony fishes, including tetrapods) and Chondrichthyes (cartilaginous fishes, including sharks, rays and chimaeras). One of its synapomorphies is the visceral endoskeleton, differentiated from preskeletal mesenchymal condensations, supporting the pharyngeal gill arches [1], [2]. There are, primitively, five branchial arches located immediately posterior to the hyoid arch, presumably the equivalent of the third to seventh visceral arches [1], [3], [4]. The posteriormost arch lies caudal to the last pharyngeal pouch and is usually smaller than the anterior arches.

Gnathostome branchial arches are usually composed of a series of articulated rods. Ventrally to dorsally, these are the unpaired and medially located basibranchials, and the paired hypobranchials, ceratobranchials, epibranchials, and pharyngobranchials [1], [5]. The basibranchials of adjacent arches are frequently fused. The ceratobranchial cartilages are serially homologous to the mandibular (Meckel's) cartilage and ceratohyal, and the epibranchial cartilages are serially homologous to the palatoquadrate and hyomandibula [5]. The identity of dorsal and ventral parts of the branchial skeleton is patterned by distinct expressions of *Dlx* genes [6], [7].

The epibranchials are the main dorsal gill arch elements. Their distal (ventral) ends articulate with the corresponding ceratobranchials while their proximal (dorsal) tips attach to the pharyngobranchials. Epibranchials may also support dermal plates for pharyngeal teeth, or develop a series of processes and flanges for the attachment of dorsal branchial muscles [8]. Ossification of the epibranchials, as well as of most branchial skeleton elements, is endochondral [9]. Their cartilaginous precursors arise early in development by condensation of undifferentiated mesenchymal cells [10], [11].

Among living gnathostomes, the visceral skeletal anatomy of Chondrichthyes most resembles the inferred primitive gnathostome pattern because every arch is similar to each other, with the posterior arches providing support for most respiratory gill surfaces (a hemibranch associated with the hyomandibula in sharks or with the pseudohyal arch in rays, plus four holobranchs on branchials arches 1–4), and because all “typical” skeletal segments are retained [10]. In chondrichthyans, at least in extant species, a discrete epibranchial 5 is not found associated with the fifth gill arch, as its primordium fuses during development [10] at least with pharyngobranchial 4, forming a complex cartilage [12], [13]. This morphology is widespread among chondrichthyans, being retained even in members of Hexanchiformes, which have one or two extra branchial arches [12].

In Osteichthyes, branchial arches 1–4 are always completely present while the fifth arch is comparatively compact with dorsal elements (epibranchials, pharyngobranchials) usually missing or smaller than the serially homologous anterior elements, which are rarely ossified when present [14]. A typical, elongate epibranchial 5 articulating with the distal tip of ceratobranchial 5 is not known in Actinopterygii. On the other hand, certain actinopterygians have a small, nodular to strip-like and generally cartilaginous piece close to the distal tips of ceratobranchials 4 and 5, although it is firmly attached to the distal extremity of ceratobranchial 4 only. This piece is usually identified as epibranchial 5 (e.g. [15]–[19]), and may be involved in supporting the crumenal organ of the Argentinoidei (Argentinoidea + Alepocephaloidea, *sensu* [20]; [21]) as well as the epibranchial organ of ostariophysans [15], [22]–[24].

Homology of the fifth epibranchial of actinopterygians and its phylogenetic origin has received previous attention [8], [15], [16], [20], [23], [25]. Most authors, likely due to its location and because a dorsal bar effectively attached to ceratobranchial 5 is missing, have uncritically identified this element in actinopterygians as epibranchial 5 (e.g. [15], [17]–[19]). Even though other authors have recognized that this piece is articulated with ceratobranchial 4 instead of with ceratobranchial 5, its homology has not been disputed (e.g. [14], [20], [23], [25], [26]). Some authors have coined new names for this element, explicitly identifying it as a neomorphic structure and implying its non-homology to epibranchial 5, absent in actinopterygians; cogent explanations for these conclusions, however, were not provided (e.g. [24], [27]–[30]). Despite these indications, the identity of the small piece associated to ceratobranchial 4 has remained open because a detailed morphological analysis, within a robust phylogenetic framework, has not been attempted until the present study.

Given the distinct topographic relationships of the element traditionally treated as the epibranchial 5 (attached to ceratobranchial 4 rather than to ceratobranchial 5 as seen in chondrichthyans), and its conspicuous morphology in several actinopterygian lineages (usually nodular instead of rod-like), we suspected that it is not part of the epibranchial series in bony fishes. To test this, we carried out a morphological and histological investigation of the dorsal elements of the branchial arches in three representatives of the Ostariophysi at different developmental stages. To evaluate the morphology and distribution of the fifth epibranchial and the nodular element that is associated to ceratobranchial 4 (hereafter referred to as “accessory element of ceratobranchial 4”), we carried out a survey of the literature and raised new data by direct examination of other gnathostomes.

## Materials and Methods

To investigate the homology of the branchial element identified as epibranchial 5 in gnathostomes, we carried out a comparative morphological analysis of its anatomy (skeleton and muscles) in adults of all major lineages, coupled with an ontogenetic study of the gill skeleton in three species of ostariophysans. To pinpoint the phylogenetic origin of the accessory element of ceratobranchial 4 and true epibranchial 5, these elements were mapped onto a gnathostome phylogeny after a review of the literature and examination of comparative material.

Developmental series of *Lophiosilurus alexandri* (LIRP 5992), *Pseudoplatystoma corruscans* (LIRP 5987), and *Prochilodus argenteus* (LIRP 5993) were obtained from the larviculture laboratory of the Hidrobiology and Hatchery Station of the municipality of Três Marias, Minas Gerais State, Brazil (Companhia de Desenvolvimento dos Vales do São Francisco e do Parnaíba, CODEVASF). Individuals were reared from natural spawning of wild specimens, in flowing water. Many specimens were collected every day during the first 10 days, then, at intervals varying from two to up to 10 days. Larvae were fixed in 10% phosphate buffered formalin for 24 to 48 hours, and then transferred to 70% ethanol. Specimens were measured to the nearest 0.1 mm notochord length (NL) for preflexion stage, and standard length (SL) for post-flexion larvae. Age is referred to days post-hatching (DPH).

Several specimens of each developmental stage were submitted to skeletal preparation following Taylor and Van Dyke [31] with some modifications (in particular, reducing acidity during Alcian Blue staining of larvae). All statements concerning chondrification and ossification were based on visual identification of elements stained with this technique, except when stated otherwise. All listed material refers to cleared and double stained specimens. Cartilage appears as blue and bone as red in both photographs and schematic drawings.

The terms ‘epal’ and ‘ceratal’ used throughout the text are adjectives applied to all parts situated dorsally (e.g. epibranchials and pharyngobranchials) and ventrally (e.g. ceratobranchials and hypobranchials) in the branchial basket, respectively.

Specimens were photographed at various magnifications with a Leica (MZ 16) stereomicroscope fitted with a Leica DC 500 digital camera connected to a PC computer. Due to reduced depth of field at great magnifications, it was necessary to take several images at different focal planes. Separate images were mounted using the open-source image processing software package CombineZP (by Alan Hadley http://www.hadleyweb.pwp.blueyonder.co.uk/), resulting in a composite image that is fully focused.

For histological analysis, fixed specimens were embedded in paraffin, cut into 5–6 µm serial sections and stained either with 1% acid Toluidine Blue, Hematoxylin and Eosin (HE), or Masson Trichrome. Sections were mounted with Permount (Fisher). Histological images were taken using a Leica microscope (DM2500), and subject to the same method cited above. All images were treated using Adobe Photoshop CS4 and Adobe Illustrator CS4 to enhance contrast and brightness.

The cladogram employed is a compilation of the phylogenetic hypotheses of Janvier [1], Carvalho [13], Gardiner et al. [32], Grande [33], Davis et al. [34], Grogan et al. [35], Stiassny et al. [36], Cloutier and Ahlberg [37], Xu and Gao [38] and Long et al. [39]. Both the cladogram and parsimony ancestral character state reconstruction were made in Mesquite [40]. We have selected relevant terminals representative of the major gnathostome clades in which information on epibranchial 5 and/or the accessory ceratobranchial elements were available. Teleost classification follows Wiley and Johnson [41]. Protacanthopterygii also follows Wiley and Johnson [41], who constrained the group to Argentiniformes (Argentinoidei + Alepocephaloidei) + Salmoniformes (Esocoidei + Osmeroidei + Salmonoidei).

To complement information in the literature on the comparative morphology and development of dorsal branchial arch elements, the following cleared-and-double stained material was examined:

### Chondrichthyes

Callorhynchidae: *Callorhinchus capensis*, ANSP 174852, 227 mm TL (total length). Carcharhinidae: *Rhizoprionodon porosus*, UFPB 1445.2, 230 mm TL. Hemiscylliidae: *Hemiscyllium ocellatum*, AMNH 38151, 213 mm TL. Pristidae: *Anoxypristis cuspidata*, AMNH 3268, 241 mm TL. Squatinidae: *Squatina californica*, AMNH 55686, 255 mm TL. Rajidae: *Raja binoculata*, AMNH 38156, 191 mm TL. Narcinidae: *Narcine brasiliensis*, UERJ 1176.4, 166 mm TL. Dasyatidae: *Taeniura lymma*, AMNH 44079, 310 mm TL. Gymnuridae: *Gymnura micrura* FMNH 89990, 146 mm TL.

### Actinopterygii

Polypteridae: *Erpetoichthys calabaricus*, MZUSP 63077, 254.2 mm SL (standard length); *Polypterus* sp., LIRP 7426, 62.5 mm SL; MZUSP 107872, disarticulated. Acipenseridae: *Acipenser fulvescens*, MZUSP 48364, 98.3 mm SL. Lepisosteidae: *Lepisosteus* sp., MZUSP 112096, 64.7 mm SL. Amiidae: *Amia calva*, MZUSP 46123, 5 specs., 31.2 to 34.9 mm SL; MZUSP 104454, 66.8 mm SL; USNM 231404, 181.1 mm SL. Hiodontidae: *Hiodon tergius*, MZUSP 28540, 81.5 mm SL; MZUSP 107933, 32.1 mm SL. Arapaimidae: *Arapaima gigas*, LIRP 4584, 120.9 mm SL; MZUSP 26083, 142.8 mm SL; MZUSP 96855, 171.8 mm SL. Osteoglossidae: *Osteoglossum* sp., MZUSP 112097, 196.6 mm SL; MZUSP 17686, 64.2 mm SL. Albulidae: *Albula vulpes*, MZUSP 10625, 101.3 mm SL. Megalopidae: *Megalops cyprinoides*, USNM 173580, 103.3 mm SL. Denticipitidae: *Denticeps clupeoides*, MZUSP 84776, 3 specs., 26.8–36.9 mm SL. Engraulidae: *Thryssa mystax*, MZUSP 112098, 124.4 mm SL. Clupeidae: *Hilsa kelee*, USNM 276407, 121.3 mm SL. Chanidae: *Chanos chanos,* ANSP 63296, 2 specs., 91.5 to 96.2 mm SL. Kneriidae: *Kneria auriculata*, ANSP 177939, 44.1 mm SL. Catostomidae: *Catostomus commersoni*, MZUSP 112099, 120.3 mm SL. Anostomidae: *Leporinus obtusidens*, LIRP 5990, 14 developmental stages, several specs. each, 3.6 mm NL (notochord length) to 77.8 mm SL. Characidae: *Brycon orthotaenia*, LIRP 5983, 10 developmental stages, several specs. each, 4.2 mm NL to 22.8 mm SL. *Salminus* sp., LIRP 5991, 4 developmental stages, several specs. each, 9.9 mm NL to 22.7 mm SL. Prochilodontidae: *Prochilodus argenteus*, LIRP 5993, 20 developmental stages, several specs. each, 3.3 mm NL to 41.4 mm SL. MZUSP 42718, 2 specs., 84.4 to 96.7 mm SL. Gymnotidae: *Gymnotus carapo*, LIRP 2126, 5 specs., 14.7 to 48.7 mm SL. *Gymnotus sylvius*, MZUSP 85947, 21 specs., 12.3 to 16.2 mm SL. Hypopomidae: *Brachypopomus* sp., LIRP 2080, 3 specs., 36.4 to 83.3 mm SL. Diplomystidae: *Diplomystes mesembrinus*, MZUSP 62595, 148.1 mm SL. Doradidae: *Franciscodoras marmoratus*, LIRP 5988, 2 specimens, 27.4 to 31.8 mm SL. Loricariidae: *Pterygoplichthys etentaculatus*, LIRP 5986, 16 developmental stages, several specs. each, 4.7 to 40.1 mm SL. *Rhinelepis aspera*, LIRP 5985, 5 specs., 18.3 to 29.7 mm SL. Pimelodidae: *Pimelodus maculatus*, LIRP 5984, 5 specs., 33.3 to 45.5 mm SL. *Pimelodus ortmanni*, LIRP 10053, 88.1 mm SL. *Pseudoplatystoma corruscans*, LIRP 5987, 14 developmental stages, several specs. each, 3.7 mm NL to 41.2 mm SL. Pseudopimelodidae: *Lophiosilurus alexandri*, LIRP 5992, 42 developmental stages, several specs. each, 6.5 mm NL to 53.2 mm SL. *Pseudopimelodus charus*, LIRP 5989, 11 specs., 12.3 to 32.1 mm SL. Esocidae: *Esox americanus*, MZUSP 112100, 2 specs., 117.6–122.2 mm SL. Salmonidae: *Salmo* sp., MZUSP 112101, 123.3 mm SL. Atherinidae: *Atherinella brasiliensis*, LIRP 1687, 2 specs., 80.2 to 101.5 mm SL. Ogcocephalidae: *Ogcocephalus vespertilio*, LIRP 4279, 77.8 mm SL. Anablepidae: *Anableps* sp., MZUSP 43103, 99.8 mm SL. Sciaenidae: *Larimus breviceps*, LIRP 1690, 2 specs., 61.7 to 66.9 mm SL; *Paralonchurus brasiliensis*, LIRP 1691, 71.8 mm SL; *Umbra limi*, MZUSP 38284, 84.6 mm SL. Phycidae: *Urophycis mystaceus*, MZUSP 40220, 196.6 mm SL.

### Sarcopterygii

Lepidosirenidae: *Lepidosiren paradoxa*, LIRP 9050, 301 mm SL; MZUSP 112102, 158.6 mm SL.

Institutional acronyms are as follows: AMNH, American Museum of Natural History, New York, USA; ANSP, Academy of Natural Sciences of Drexel University [formerly Academy of Natural Sciences], Philadelphia, USA; FMNH, Field Museum of Natural History, Chicago, USA; LIRP, Laboratório de Ictiologia de Ribeirão Preto, Faculdade de Filosofia, Ciências e Letras de Ribeirão Preto, Universidade de São Paulo, Brazil; MZUSP, Museu de Zoologia da Universidade de São Paulo, São Paulo, Brazil; UERJ, Universidade do Estado do Rio de Janeiro, Rio de Janeiro, Brazil; UFPB, Departamento de Sistemática e Ecologia, Universidade Federal da Paraíba, João Pessoa, Brazil; USNM, National Museum of Natural History, Smithsonian Institution, Washington, D.C., USA.

## Results and Discussion

### Formation of epibranchials and accessory element of ceratobranchial 4 in *Prochilodus argenteus* (Characiformes, Prochilodontidae)

Epibranchial cartilages are first seen at the 5.6 mm SL stage (4 DPH), arising at the distal ends of the respective ceratobranchials. At this stage, the branchial basket is at the beginning of its formation and most structures are weakly stained. The most evident elements are the cartilaginous bars of ceratobranchials 1–5. Along the midline, there is a single cartilaginous bar, the anterior copula, with no segmentation. The cartilages are not completely formed, but are already isolated and not fused to the ceratobranchial or to the anterior copula. Epibranchial cartilages 1–4 are present at this stage, and are also poorly stained. They are located dorsal to the ceratobranchials, chondrifying at the dorsolateral end of the respective ceratobranchial cartilages. Epibranchial 4 is the broadest; epibranchials are progressively smaller anteriorly.

**Figure 1:**
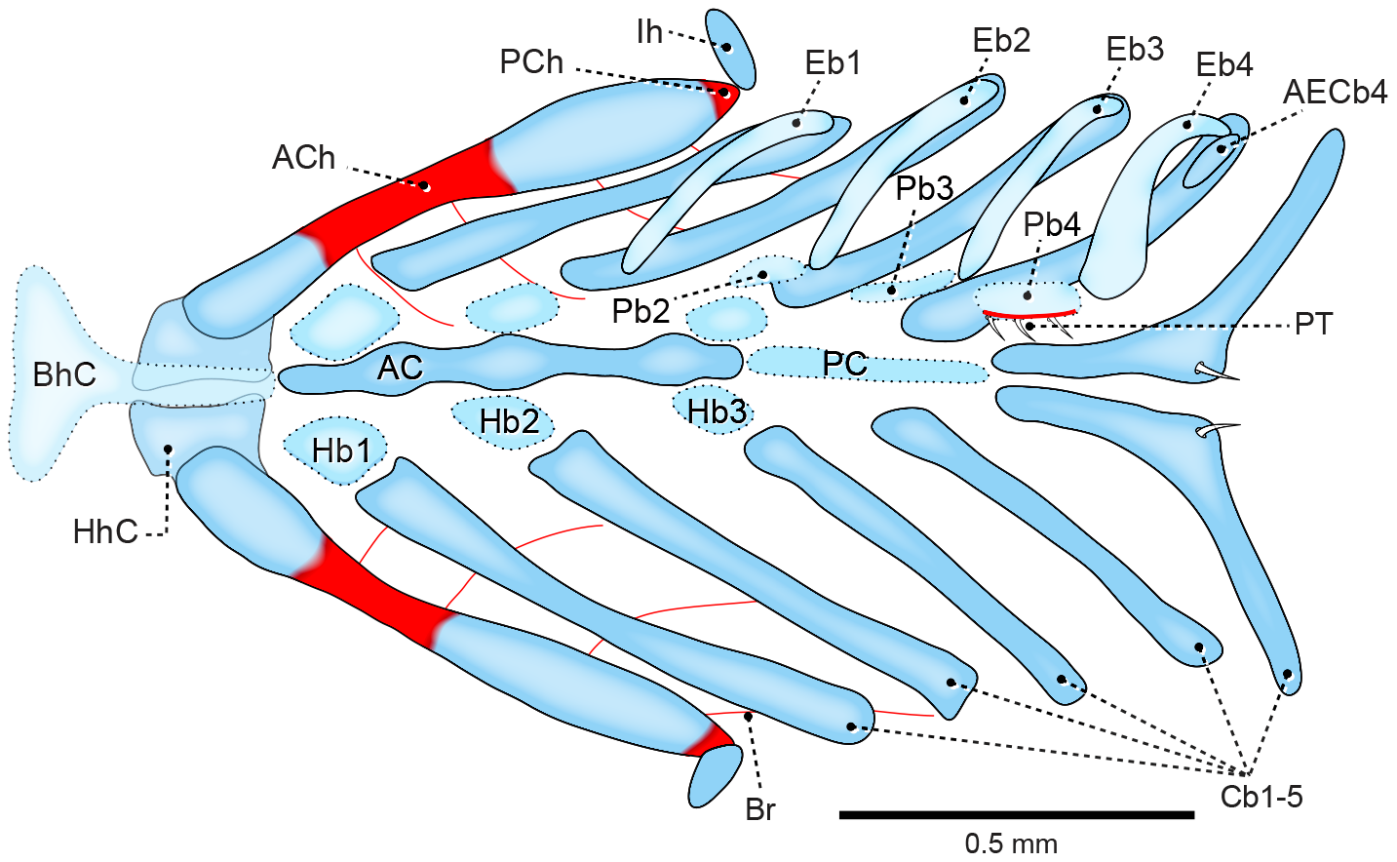
Branchial basket of *Prochilodus argenteus* (10.9 mm SL, 7 DPH–LIRP 5993). Dorsal view (anterior to left). Dorsal elements not represented on left side. Abbreviations: ACh, anterior ceratohyal; AC, anterior copula; AECb4, accessory element of ceratobranchial 4; BhC, basihyal cartilage; Br, branchiostegal ray; Cb1–5, ceratobranchials; Eb1–4, epibranchials; Hb1–3, hypobranchials; HhC, hypohyal cartilage; Ih, Interhyal; Pb2–4, pharyngobranchials; PCh, posterior ceratohyal; PC, posterior copula.

At the 10.9 mm SL stage (7 DPH; **Figure 1**), the elements of the branchial basket are fully formed. Hypobranchials 1–3 remain weakly calcified, as well as the newly formed posterior copula. The accessory element of ceratobranchial 4 emerges as a tiny structure at the dorsolateral end of ceratobranchial 4, close to epibranchial 4.

**Figure 2:**
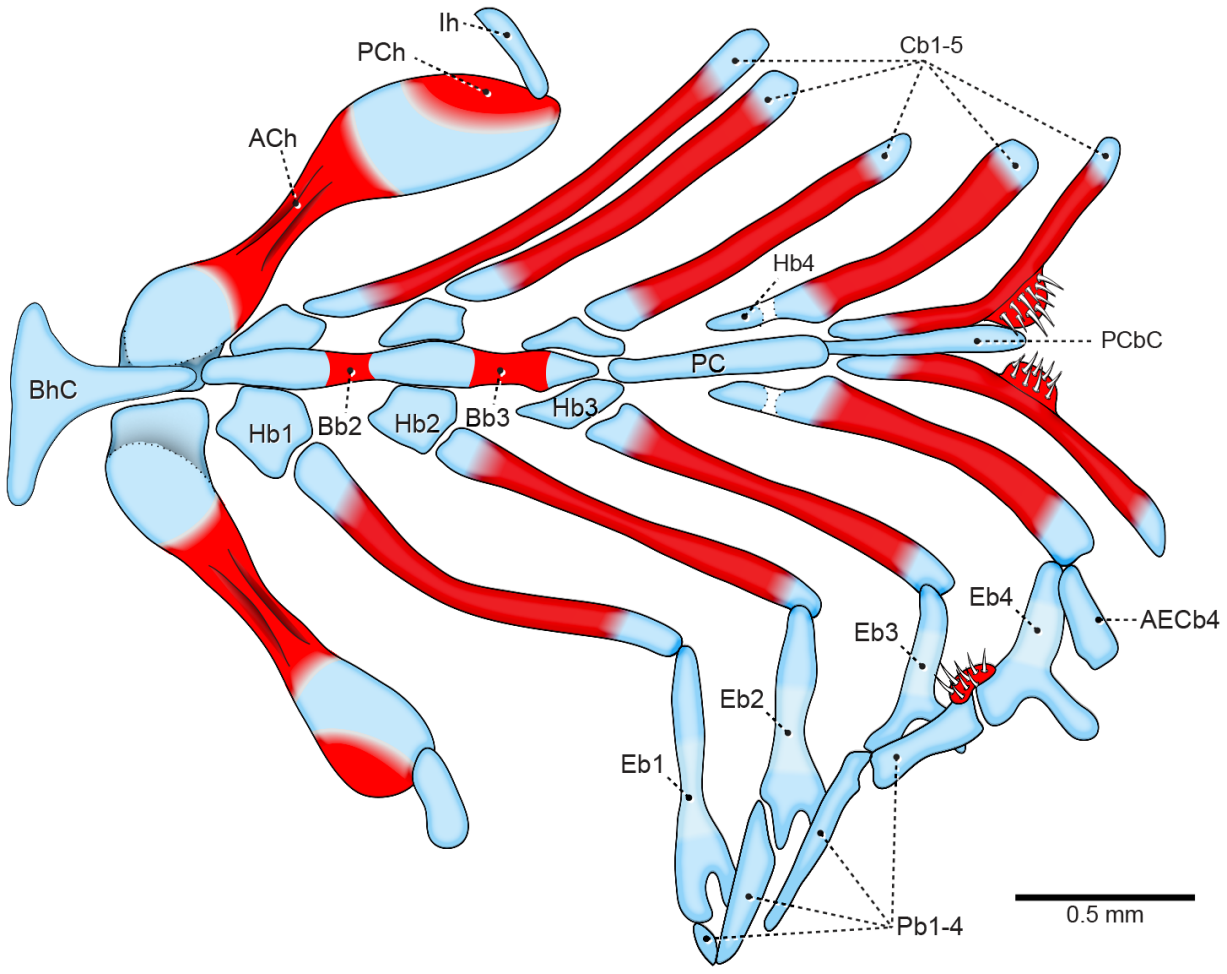
Branchial basket of *Prochilodus argenteus* (12.9 mm SL, 9 DPH–LIRP 5993). Dorsal view (anterior to left). Dorsal elements not represented on right side and unfolded on left side. Abbreviations: ACh, anterior ceratohyal; AECb4, accessory element of ceratobranchial 4; Bb2–3, basibranchials; BhC, basihyal cartilage; Cb1–5, ceratobranchials; Eb1–4, epibranchials; Hb1–3, hypobranchials; Ih, interhyal; Pb1–4, pharyngobranchials; PCbC, posterior ceratobranchial cartilage; PCh, posterior ceratohyal; PCp, posterior copula;

At the later stage of 12.9 mm SL (9 DPH), epibranchial cartilages 1–4 develop anterodorsally pointed uncinate processes. The accessory element of ceratobranchial 4 remains a slender cartilaginous bar, but extends to almost reach the uncinate process of epibranchial 4 (**Figure 2**). Ossification of the epibranchial series begins only at the 14.1 mm SL stage (12 DPH), maintaining the pattern of endochondral ossification, which starts at the middle of the cartilage and extends towards the extremities. The accessory element of ceratobranchial 4 never ossifies.

### Formation of epibranchials and accessory element of ceratobranchial 4 in *Lophiosilurus alexandri* (Pseudopimelodidae, Siluriformes)

At 7.7 mm SL (3 DPH), the branchial basket is not yet completed formed, still lacking some ventral elements. The branchial basket has along its ventral midline a cartilaginous structure, but no independent hypobranchial or basibranchial cartilages are evident. The cartilaginous ceratobranchials 1–4 are well defined, staining in deep blue. The posteriormost ceratobranchial 5 is also present but is weakly calcified, indicating the anteroposterior sequence of the appearance of these elements. At this stage, all four cartilaginous epibranchials (1–4) are already formed, but the accessory element of ceratobranchial 4 cannot yet be detected. Epibranchial cartilages chondrify at dorsolateral tips of the corresponding cartilaginous ceratobranchial 1–4. Epibranchial cartilages 1–3 are short, narrow and cylindrical, and poorly stained, chondrifying anteromedially. They remain rectilinear until ossification initiates. Epibranchial 4 is the largest and most intensely calcified of the epibranchial series. It has a triangular shape, with the anteromedial end twice as broad as its lateroposterior portion, which articulates with ceratobranchial 4.

**Figure 3:**
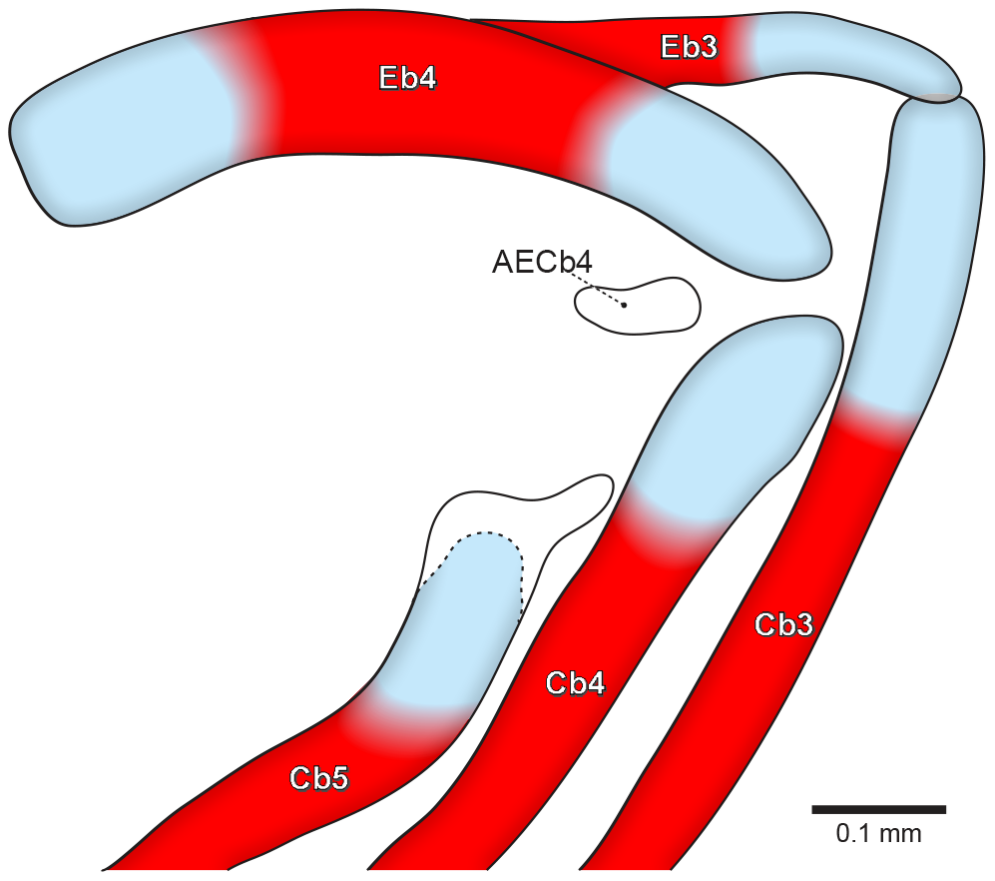
Posterior portion of the caudalmost branchial arches of *Lophiosilurus alexandri* (11.7 mm SL, 8DPH–LIRP 5992). Right side, in posterior view. Abbreviations: AECb4, accessory element of ceratobranchial 4; Cb3–5, ceratobranchials; Eb3–4, epibranchials.

The accessory element of ceratobranchial 4 is first visible at 11.7 mm SL (8 DPH) (**Figure 3**). It chondrifies dorsally and posteriorly at the lateral tip of ceratobranchial 4 cartilage, adjacent to the epibranchial 4 cartilage. At this stage, it is very delicate and unstained, being only possible to observe it by changing the light angle under the stereomicroscope.

In the stage in which the accessory element of ceratobranchial 4 appears (11.7 mm SL, 8DPH), the entire branchial basket is already completely formed, with all elements well defined and most of them already ossified, except the basi- and hypobranchials.

Ossification of epibranchials 1–4 begins at 11.2 mm SL (8 DPH) at the midlength of each cartilaginous rod and spreads in all directions. Epibranchial 3 has an uncinate process lying dorsal to its medial portion. Accessory element of ceratobranchial 4 does not ossify and remains short, slender and rounded in adults.

### Formation of epibranchials and accessory element of ceratobranchial 4 in *Pseudoplatystoma corruscans* (Pimelodidae, Siluriformes)

The first signs of epibranchial cartilages are visible at the 3.8 mm SL stage (3 DPH) when the branchial basket is just at the beginning of its formation; however, some weakly stained elements can be observed. Branchial basket ventral midline has a single cylindrical cartilaginous structure, with no sign of segmentation of hypobranchial and basibranchial cartilages. Ceratobranchial cartilages 1–4 are already formed, albeit weakly stained. Posteriormost ceratobranchial 5 cartilage is also present but is not stained, observed only by changing the light angle under the stereomicroscope. At this stage, epibranchial cartilages 1–4 are present, dorsal to the ceratobranchials. They chondrify at the dorsolateral tips of ceratobranchial 1–4 cartilages, respectively. Epibranchial cartilages 1–2 are very tiny, narrow, poorly stained, and very difficult to detect. Epibranchial 2 cartilage is slightly more evident, having the same shape as the anterior epibranchial. Epibranchial 4 cartilage is the largest and most intensely stained of this series. It is triangular, with the anteromedial portion being twice as robust as its lateroposterior aspect (which articulates with ceratobranchial 4).

**Figure 4:**
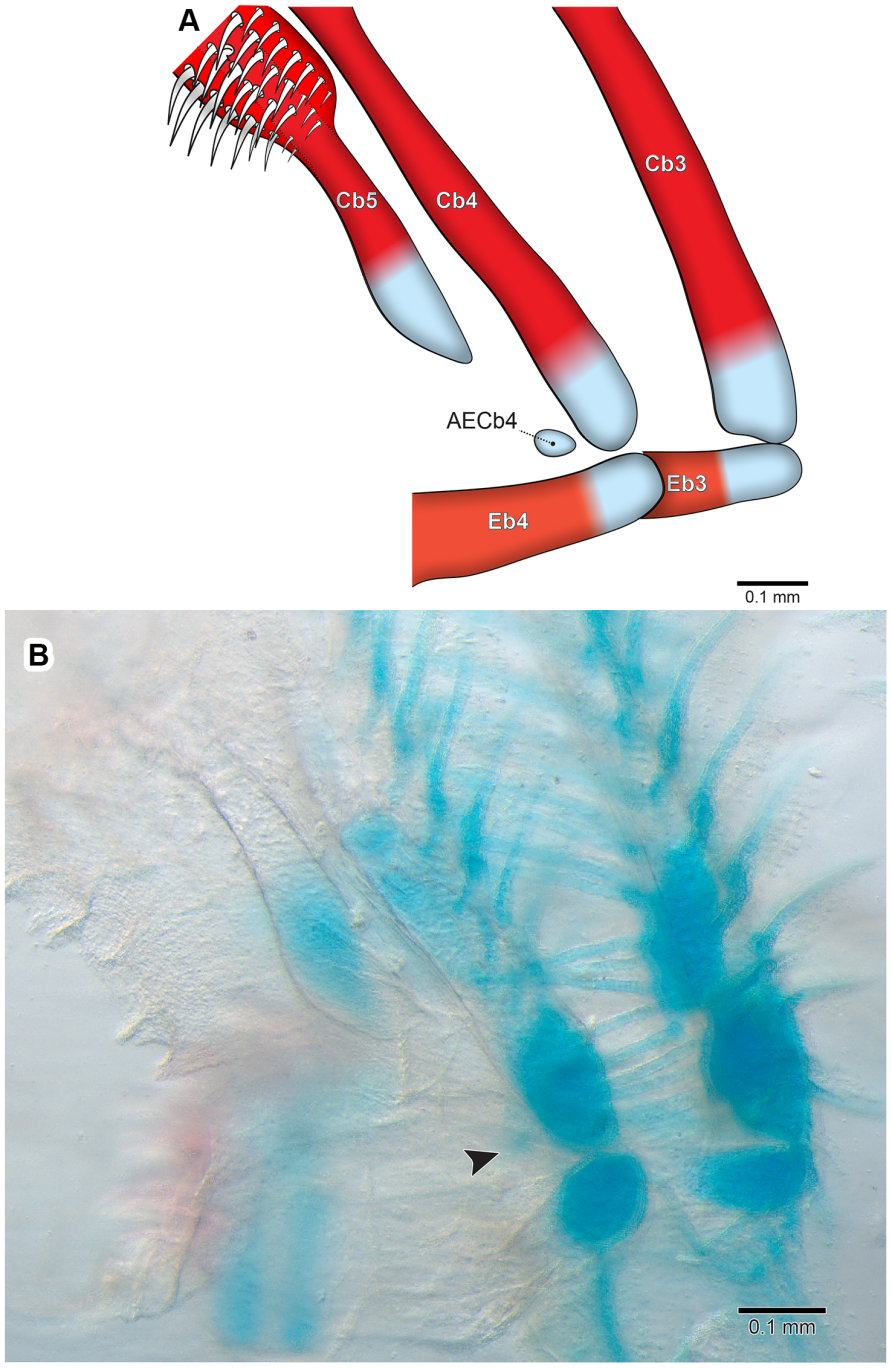
Posterior portion of the last right side branchial arches of *Pseudoplatystoma corruscans* (21.4 mm SL, 26 DPH–LIRP 5987). Posterior view. A, schematic drawing; B, photograph of a c&s specimen. The bones are not stained (in red) due to the very initial stage of perichondral ossification. Arrowhead points to the accessory element of ceratobranchial 4. Abbreviations: AECb4, accessory element of ceratobranchial 4; Cb3–5, ceratobranchials; Eb3–4, epibranchials.

Accessory element of ceratobranchial 4 is only observed at 21.4 mm SL (26 DPH) (**Figure 4**). It chondrifies posteriorly at the lateral tip of ceratobranchial 4 cartilage next to epibranchial 4 (which is dorsally located), and is very delicate and poorly stained. At this stage, the entire branchial basket is already completely formed, with all elements well defined and recently ossified (a process that initiated in the branchial basket at 8DPH).

Ossification of epibranchials 1–4 begins at the 11.1 mm SL stage (19 DPH) at the middle of each cartilage and spreads in all directions. In a later stage (28 DPH), epibranchial 3 has an uncinate process lying dorsal to its medial portion. Accessory element of ceratobranchial 4 is short, slender, and rounded, and remains cartilaginous in adults.

### Histological investigation

**Figure 5:**
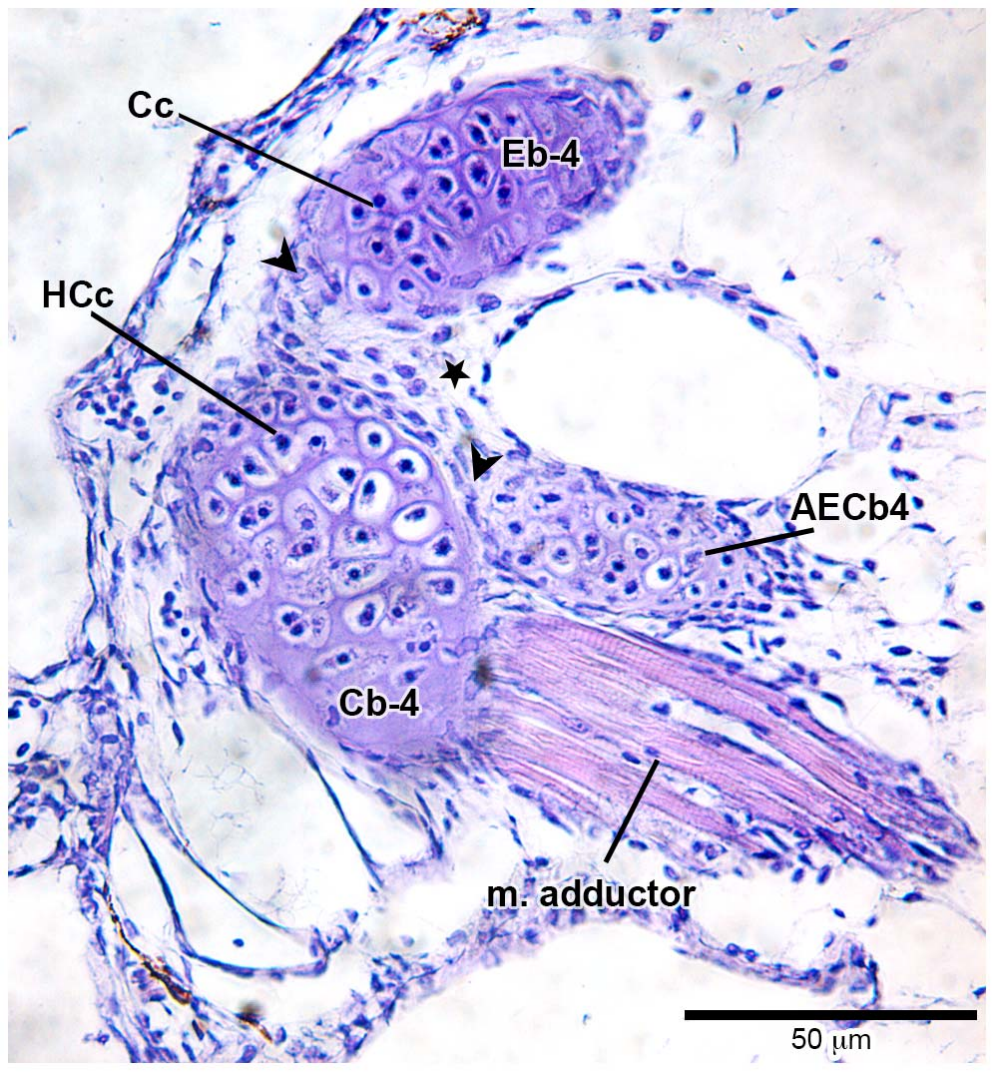
Histological section at level of the distal tip of fourth branchial arch of *Lophiosilurus alexandri* (11.7 mm SL, 8 DPH–LIRP 5992). The articulation area of ceratobranchial 4 with epibranchial 4, evidencing the initial stage of rising of the accessory element of ceratobranchial 4. Abbreviations: AECb-4, accessory element of ceratobranchial 4; Cb-4, ceratobranchial 4; Cc, chondrocyte; Eb-4, epibranchial 4; HCc, hypertrophied chondrocyte; m. adductor, muscle adductor of the arch 4; arrowhead, chondroblasts layer (flatten cells); star, mesenchymal (undifferentiated) cells. Magnification 400x.

**Figure 6:**
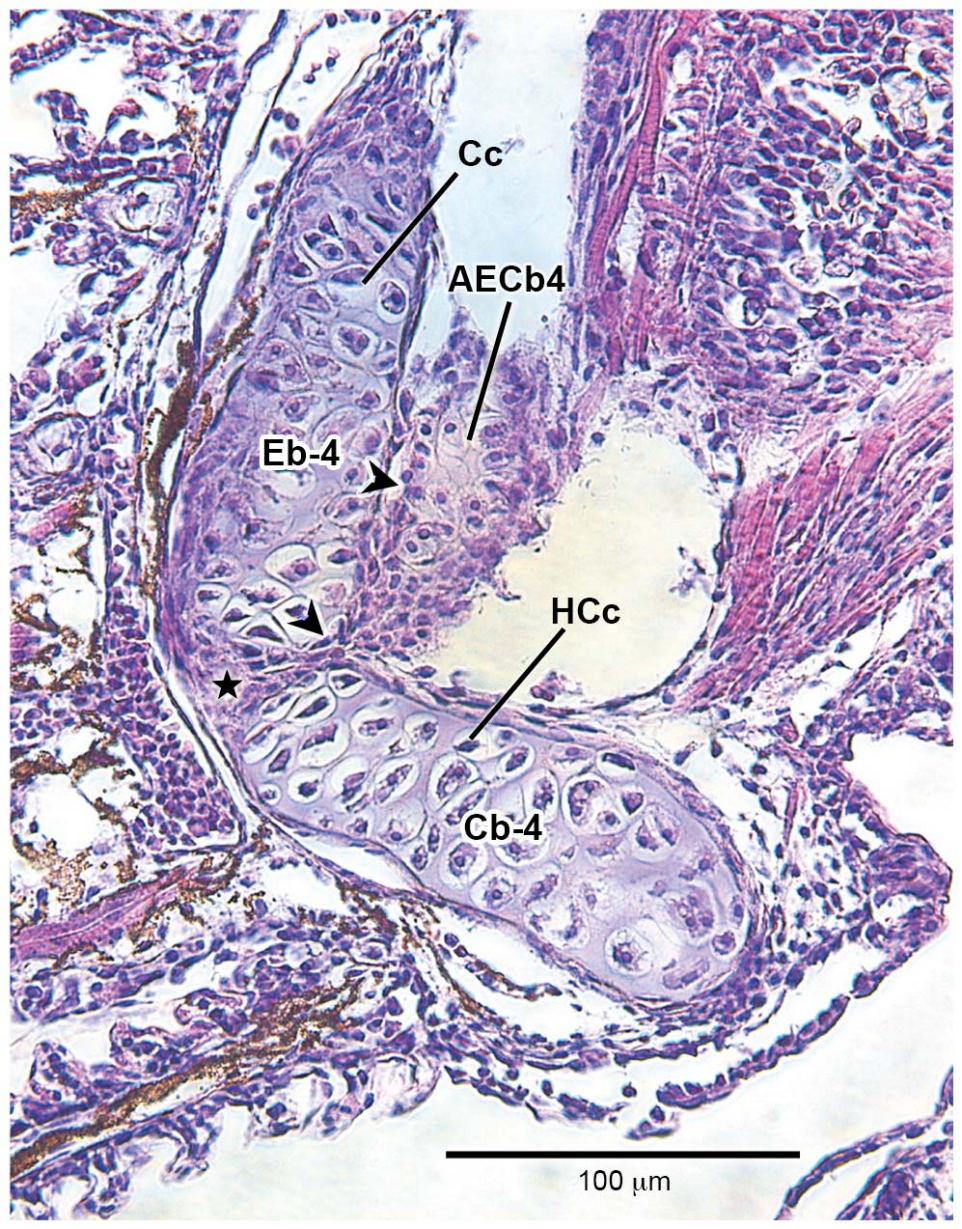
Histological section at level of the distal tip of fourth branchial arch of *Prochilodus argenteus* (12.9 mm SL, 9 DPH–LIRP 5993). The articulation area of ceratobranchial 4 with epibranchial 4, evidencing the initial stage of rising of accessory element of ceratobranchial 4. Abbreviations: AECb-4, accessory element of ceratobranchial 4; Cb-4, ceratobranchial 4; Cc, chondrocyte; Eb-4, epibranchial 4; HCc, hypertrophied chondrocyte; arrowhead, chondroblast layer (flatten cells); star, mesenchymal (undifferentiated) cells. Magnification 400x.

**Figure 7:**
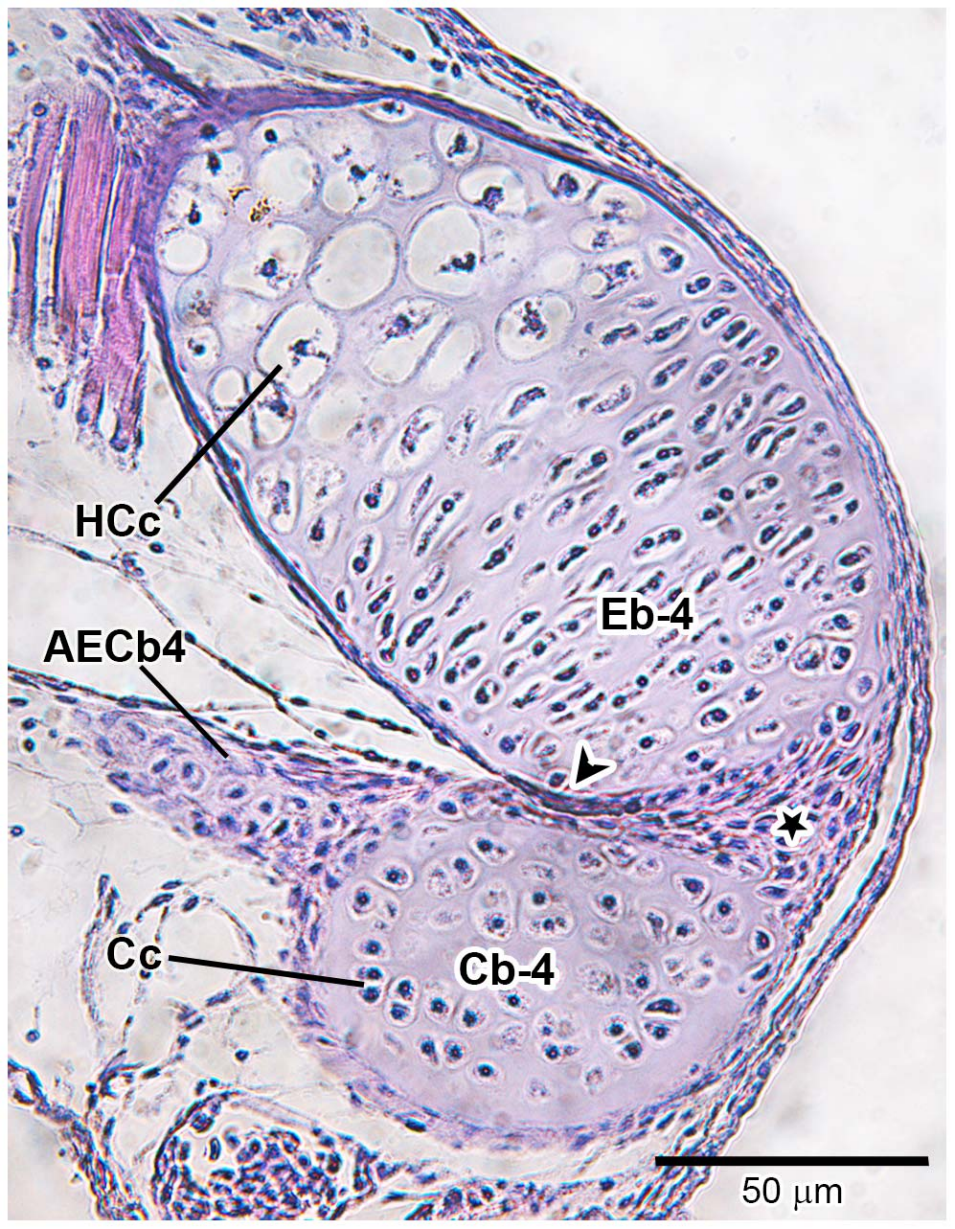
Histological section at level of the distal tip of fourth branchial arch of *Pseudoplaystoma corruscans* (21.4 mm SL, 26 DPH–LIRP 5987). The articulation area of ceratobranchial 4 with epibranchial 4, evidencing the initial stage of rising of accessory element of ceratobranchial 4. Abbreviations: AECb-4, accessory element of ceratobranchial 4; Cb-4, ceratobranchial 4; Cc, chondrocyte; Eb-4, epibranchial 4; HCc, hypertrophied chondrocyte; arrowhead, chondroblast layer (flatten cells); star, mesenchymal (undifferentiated) cells. Magnification 400x.

Histological sections of the species examined in the present study, at the initial stage of formation of accessory element of ceratobranchial 4 (which never ossifies, even in adults), reveals how close the association is between the epibranchial 5 cartilage and posterolateral tip of ceratobranchial 4; this is the region of the joint between ceratobranchial 4 and epibranchial 4. In the histological sections (*Lophiosilurus*, **Figure 5**; *Prochilodus*, Figure 6; *Pseudoplatystoma*, Figure 7) it is clear that both cerato- and epibranchial 4, despite their close proximity, have an independent layer of cartilage- forming cells (chondroblasts, see arrowhead in Figures 5, 6, 7) separated by a mesenchymal cell layer (indicated by a star), which originates the chondroblasts. Then, as chondroblasts divide, cells migrate inwardly and differentiate into the definitive cartilage-forming cells (chondrocytes), which mature, hypertrophy and die (apoptosis; Figures 5, 6, 7).

However, a close look at the cartilaginous accessory element of ceratobranchial 4 at the very beginning of its formation shows that there is an intimate connection with ceratobranchial 4. This connection is so “tight” that both structures share the same single-cell layer of chondroblasts; the chondrocytes of both accessory element of ceratobranchial 4 and ceratobranchial 4 proper are derived from the same chondroblastic layer.

### Comparative ontogeny of epibranchial 5 and accessory element of ceratobranchial 4

Even though our sample of ontogenetically investigated species is not representative of gnathostome diversity, the patterns of chondrification and ossification encountered are highly compatible with each other and with those described for other fishes. In the three species examined, all epibranchials 1–4 appear early in development, at 5.6 mm SL (4 DPH) in *P. argenteus*, 7.7 mm SL (3 DPH) in *L. alexandri*, and 3.8 mm SL (3 DPH) in *P. corruscans*. This pattern, in which all four epibranchials appear early in development either simultaneously or within a short time interval, has also been reported for species of other ostariophysan lineages, such as Gonorynchiformes (Chanidae [42], [43]), Cypriniformes (Cyprinidae [44]–[48], Catostomidae [49]), Characiformes (Characidae [26], [50]), and Siluriformes (e.g. Pangasiidae and Schilbidae [51]; Ariidae and Plotosidae [52]; Bagridae [53]; Clariidae and Heteropneustidae [54]; Clariidae [55], [56]; Callichthyidae [57]; Loricariidae [58]), as well as in a representatives of the closely related Clupeiformes (Clupeidae [59]).

On the other hand, in the three species examined, accessory element of ceratobranchial 4 appears in a very late stage, when the entire branchial basket is already completely formed, with all ceratal and epal elements well defined, and ossifying relatively late at 10.9 mm SL (7 DPH) in *P. argenteus*, at 11.7 mm SL (11 DPH) in *L*. *alexandri*, and 21.4 mm SL (26 DPH) in *P. corruscans*. Comparison with data from the literature is difficult in this case, since most authors usually do not mention the accessory element of ceratobranchial 4. However, comparative data from papers reporting the development of this element is, again, compatible with the pattern we observed (e.g. Gonorynchiformes [43]; Cypriniformes [49]).

In chondrichthyans, the cartilage attached to the distal extremity of ceratobranchial 5, which we identify as the true epibranchial 5, appears immediately after the appearance of epibranchial 4 (e.g. fig. 4 in [10]; [11], [60]). This structure develops as an independent cartilage as opposed to detaching from a cartilaginous or pre-chondrogenic precursor, as for the accessory element of ceratobranchial 4 (see our histological data above). Therefore, from an ontogenetic perspective, we can confidently say that epibranchial 5 in chondrichthyans is, in fact, part of the epibranchial series–it is serially homologous to the anterior dorsal elements that are attached to the anterior ceratobranchials.

During the emergence of epibranchials 1–4 in all taxa examined (Figures 5, 6, 7), it was clear that the ceratal and epal primordial cartilages of each branchial arch have their own mesenchymal cell layers, even in regions where corresponding cerato- and epibranchials are in close proximity. In addition, cartilages of each arch, such as ceratobranchial 4 and epibranchial 4, have an independent chondroblastic cell layer. This indicates that each element arises independently from its own primordial mesenchymal condensation, the first step of the chondrification process (see [2], [5], [61] for details). In contrast, accessory element of ceratobranchial 4 arises and develops in intimate connection with ceratobranchial 4, lacking its own chondroblastic layer (it shares the same chondroblastic layer with ceratobranchial 4). This means it does not have a completely autogenous origin. Given that early in ontogeny the layers of chondroblasts are shared between ceratobranchial 4 and the nodular cartilage attached to it (accessory element of ceratobranchial 4), it is suggested the latter is an outgrowth of the chondroblastic layer located at the distal end of the former, which later becomes partially independent with its own cartilaginous matrix.

Unfortunately, to our knowledge, there is no comparable data on the microanatomy of these structures in other fishes. However, as shown above, because the morphology and topographic relationships of the accessory element of ceratobranchial 4 in actinopterygians is virtually the same as those described above, it is reasonable to assume that they are formed by a similar ontogenetic process.

### Myological evidence bearing on the presence of epibranchial 5 in living gnathostomes

Further evidence that the accessory element of ceratobranchial 4 is not homologous to epibranchial 5 derives from myology. The presence of branchial *levator* muscles is a synapomorphy of Osteichthyes [8], [62]. Actinopterygians typically have four (branchial) *levatores externi* muscles, originating from the ventral surface of neurocranium and each inserting onto a corresponding epibranchial, innervated by the glossopharyngeal and vagus nerves. If the accessory element of ceratobranchial 4 of actinopterygians is a true epibranchial 5, it could have accommodated the insertion of the *levator externus* 5. However, this muscle is missing and the accessory element of ceratobranchial 4 sometimes provides an attachment site for a small part of *levator externus* 4 [63]. Among living osteichthyans a *levator externus* 5 is present in Actinistia (*Latimeria*) and Dipnoi (*Neoceratodus*) only [8], both of which, however, lack the accessory element of ceratobranchial 4 and epibranchial 5. In these taxa this muscle is inserted directly on non-muscular esophageal tissue just posterior to the gill arches or on the external surface of the anocleithrum, respectively [8].

Muscles associated with the dorsal aspect of the posterior branchial arches in chondrichthyans (in particular the *cucullaris*) are innervated by the glossopharyngeal and vagus nerves [M. Soares, pers. comm.] as are the *levatores externi* muscles of actinopterygians. However, in chondrichthyans, the *cucullaris* (or *cucullaris superficiali*s) originates from the dorsal fascia of the cranial portion of the epaxial body musculature and extends ventrocaudally to insert (in elasmobranchs) on the last epibranchial (or the complex structure in that position) as well as on the scapular portion of the pectoral girdle ([12], fig. 1C]; in chimaeras, insertion is solely on the shoulder girdle; ([64] figs. [38], [40], [41], [43]). In chimaeras, however, fibers of the *cucullaris profundus* originate on the otic capsule of the neurocranium and insert on separate posterior pharyngobranchials (or on the fused composite posterior cartilage [64], [65]), and may be homologous to the *levatores externi* of actinopterygians, but this is only tentative. The gill arches in chimaeras are situated more anteriorly in comparison to elasmobranchs (more underneath the braincase), and muscles bridging the neurocranium and gill arches are more easily compared. It is more difficult to hypothesize that the *cucullaris superficialis* is homologous to the *levatores externi* of actinopterygians (even though innervated by the same nerves, these are differently arranged, and these muscles are topographically very distinct). The *subspinalis* muscle originates on the posterior aspect of the neurocranium and inserts on the first or second pharyngobranchial in many chondrichthyans (including chimaeras), but also has no apparent relation to the *levatores externi* of actinopterygians.

Evidence for a separate (true) epibranchial 5, therefore, does not derive from associated muscles that are similarly innervated or positioned in gnathostomes, given that muscles are difficult to compare in this manner between chondrichthyans and osteichthyans. We note that the true epibranchial 5 is present in chondrichthyans irrespective of any possible homology between the *cucullaris* or *subspinalis* of chondrichthyans with osteichthyan dorsal branchiomeric muscles.

### Critical review of the distribution of epibranchial 5 and accessory element of ceratobranchial 4 across major gnathostome clades

**Figure 8:**
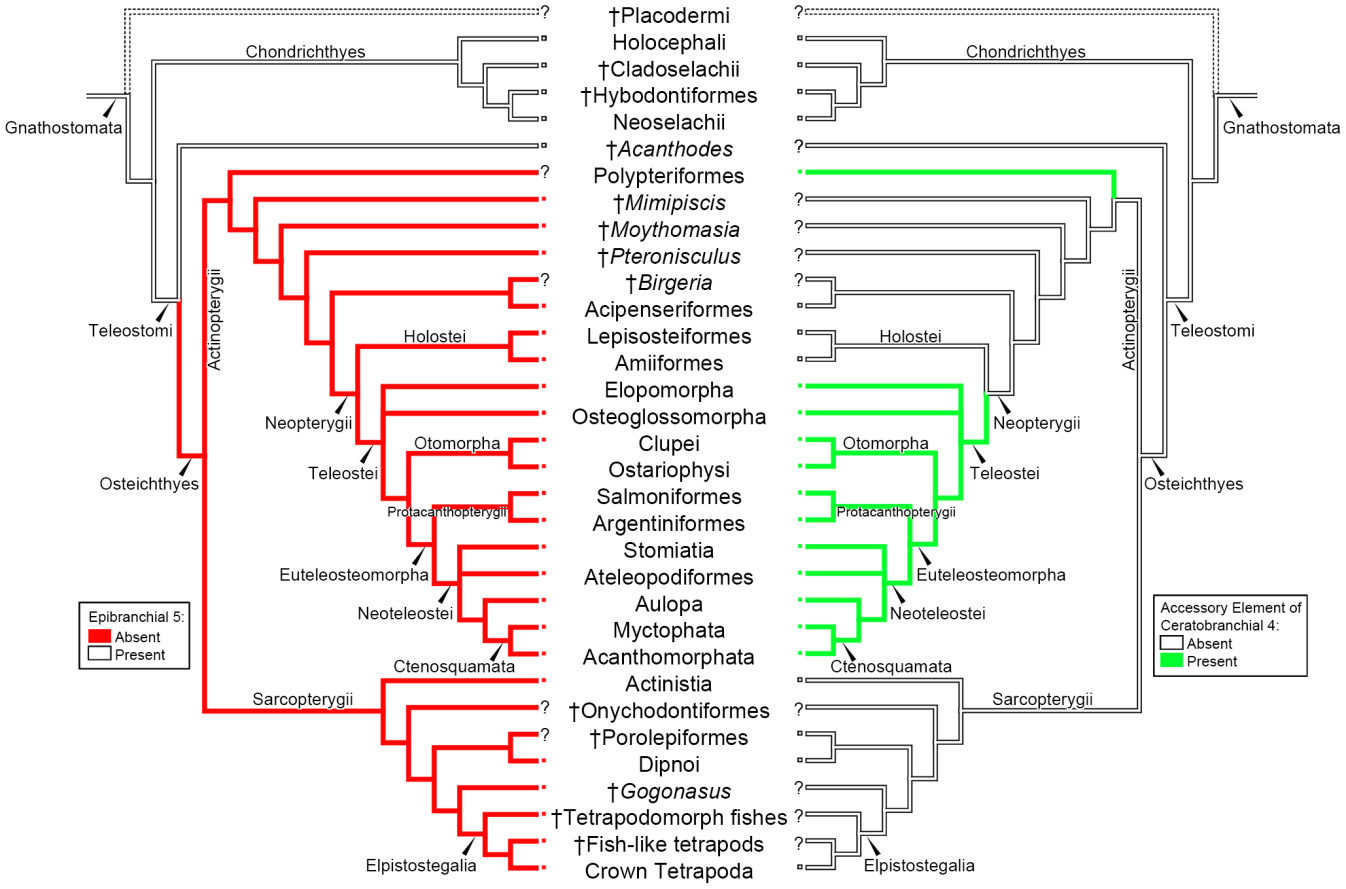
Cladogram showing the relations of the Gnathostomata taxa discussed in the present study, as well as the higher groups they represent. A parsimony ancestral character state reconstruction was made for the accessory element of ceratobranchial 4 and the epibranchial 5. Missing [?] data are indicates at the terminal tips; solid and open squares denote presence or absence of the character, respectively. General relationships were based on [1], [13], [32]–[37].

Reliable data on the presence or absence of epibranchial 5 and accessory element of ceratobranchial 4 in most gnathostomes are scant, precluding a fuller understanding of their evolution. Accessory elements of ceratobranchials are usually small and cartilaginous, and therefore frequently overlooked in descriptions and illustrations of extant fishes, sometimes due to imperfectly cleared and stained specimens. Another issue is that most basal lineages of gnathostomes are only known from incompletely fossilized specimens, as gill arches are structurally fragile and rarely preserved complete and articulated. To complicate matters, gill arches of fossils are usually covered by more robust external parts of the skeleton (e.g. the dermal opercular cover); hence, the arrangement and composition of the gill arches in stem gnathostomes are poorly known. Figure 8 depicts phylogenetic relationships among major gnathostome lineages [66], [67], with our interpretation of the evolution of epibranchial 5 and accessory element of ceratobranchial 4. Below, we comment on the occurrence and morphological variation of these characters among major groups.

#### †Placodermi

This group is usually considered monophyletic (e.g. [1], [68]–[71]) but this was recently challenged (e.g. [72]), even though in a context of acanthodian monophyly and basal gnathostome relationships wherein placoderms were not the primary focus. Discussion of dorsal elements of the posterior branchial arches would be greatly enriched if these features were preserved in placoderms. However, the posterior branchial arches are unknown with much certainty in this group, although five branchial arches are suspected to have been present [1], [73] [P. Janvier and D. Goujet, pers. comm.]. Some branchial elements have been reported in the ptyctodont †*Ctenurella* but these are scattered, impeding their proper identification [D. Goujet, pers. comm.]. More substantial data exist only for the rhenanid †*Gemuendina* [74], in which x-ray radiographs of the branchial basket *in situ* reveal four branchial arches with robust ceratobranchials, but epibranchials were not identified (nor was a fifth gill arch, which may nonetheless have existed). According to D. Goujet [pers. comm.], the dorsal contact of the branchial basket with the braincase in placoderms was through two articular areas on the posterior postorbital process by means of dorsal branchial elements (pharyngo- or epibranchials).

#### †Acanthodii

Members of this group, among the earliest gnathostome lineages, are known to have a mosaic of features common to the main basal lineages of gnathostomes, so that acanthodians are either recognized as closely related to Chondrichthyes [3], [75] or Osteichthyes [1], [76], [77], and have even been considered allied to certain placoderms [78]. Although †Acanthodii is usually regarded as monophyletic [1], [79], a recent phylogenetic analysis of acanthodians and basal chondrichthyans and osteichthyans has split them at the gnathostome base, with some acanthodian lineages positioned closer to the clade Chondrichthyes + Osteichthyes while the remaining lineages are closer to either Chondrichthyes or Osteichthyes [34].

The Permian †*Acanthodes bronni* [3], [14], [77] is the only known acanthodian with a somewhat complete branchial basket. Until recently, the presence and shape of an epibranchial 5 in †*A. bronni* was not conclusive, due to the fragmentary nature of the fossils. Reconstructions of the branchial basket of †*A. bronni* have depicted a possible rod-like epibranchial 5, similar to the anterior serially homologous elements [1], [3], [14], [75], [77], [78], [80], corresponding to a true epibranchial 5. This issue was recently enlightened by Davis et al. [34] who presented a photograph of the fossilized head of an †*Acanthodes* specimen in lateral aspect showing an unequivocal, elongate and slender epibranchial 5 that is shaped similarly to the anterior epibranchials (see their fig. 12e in supplementary data).

**Figure 12:**
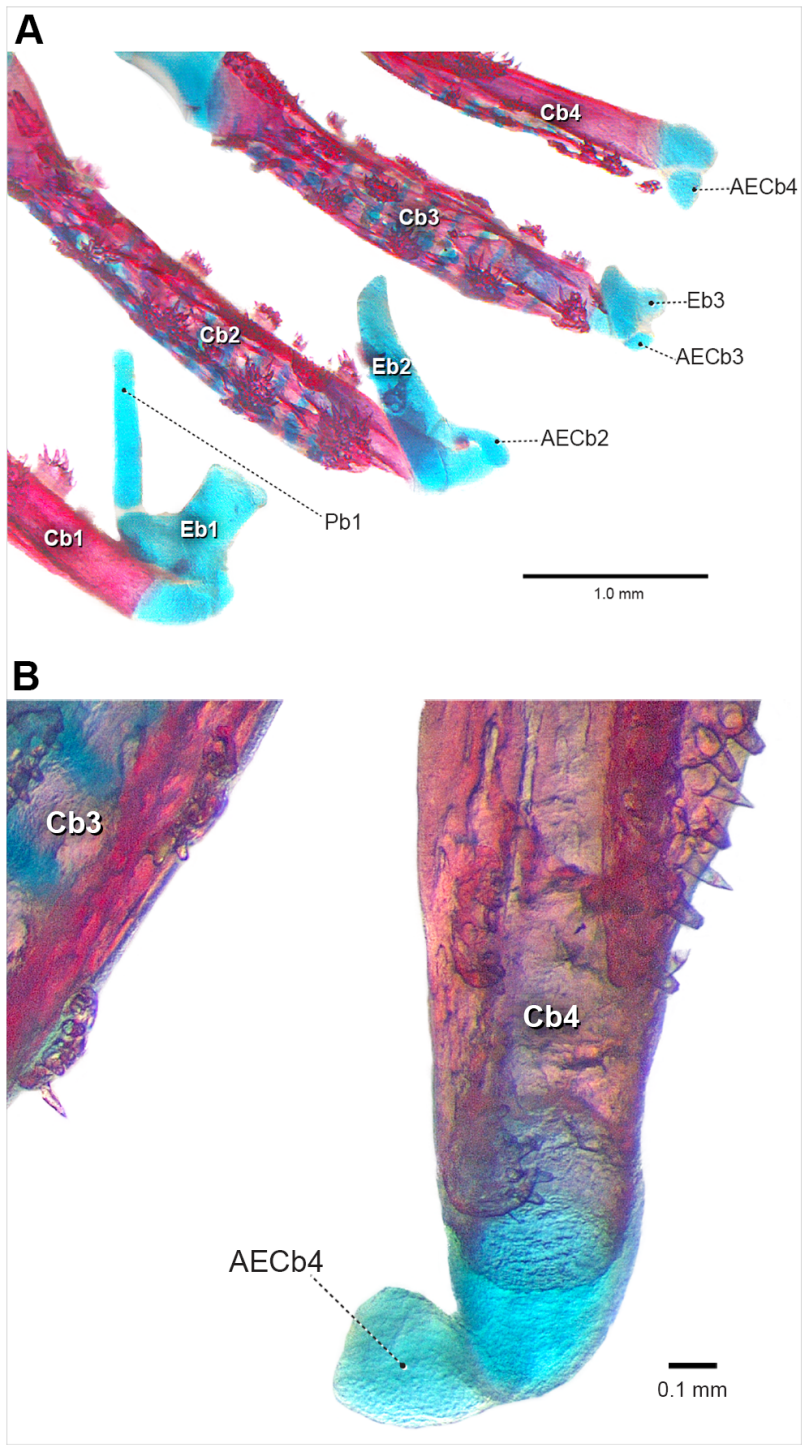
Detail of accessory elements in polypteriforms. A, distal tips of branchial arches of *Polypterus* sp. (62.5 mm SL, LIRP 7426); B, distal tips of ceratobranchial 4 of *Erpetoichthys calabaricus* (254.2 mm SL, MZUSP 63077). Abbreviations: AECb, accessory elements of ceratobranchials; Cb1–4, ceratobranchials; Eb1–3, epibranchials; Pb1, pharyngobranchial.

†*Acanthodes*, however, is a very late (Permian) acanthodian, and may not be representative of the general (stem) acanthodian condition (a difficult prospect given the possible non-monophyly of acanthodians anyway). Brazeau [81] described in detail the morphology of †*Ptomacanthus*, a much earlier acanthodian (Early Devonian), including aspects of its gill arches. Dorsal elements are depicted, and it is believed that epibranchials were indeed present as slender, rod-like elements; the presence of a fifth epibranchial, however, is still speculative (indeed of the entire fifth arch) [M. Brazeau, pers. comm.]. A similar condition is found in the Late Devonian †*Halimacanthodes*, which may be closely allied to †*Acanthodes* in the phylogeny of Davis and collaborators [John Long, pers. comm.]. Four epibranchials are clearly shown in this genus [82], and epibranchial 5 is considered likely to have been present [John Long, pers. comm.]. These observations reinforce that a fifth epibranchial was present in stem-group acanthodians.

The above data highlight that without further information on placoderm posterior gill arches the fifth epibranchial can be interpreted as either a separate derivation for acanthodians and chondrichthyans, or a character uniting chondrichthyans and teleostomes (and secondarily lost in osteichthyans). According to the phylogenetic hypothesis of Davis et al. [34], †*Acanthodes* is considered a stem osteichthyan, suggesting that a plesiomorphic true epibranchial 5, with a rod-like aspect, could also be retained among other older osteichthyans, such as in the Early Devonian genera †*Cassidiceps*, †*Dialipina*, †*Euthacanthus*, †*Ischnacanthus*, and †*Ligulalepis* [34], which lack branchial arch data. No accessory elements attached to the distal portion of ceratobranchials are so far known in †Acanthodii. However, accepting the homology of the morphologically and topologically similar epibranchials 1–4 in †*Acanthodes* and Chondrichthyes implies that epibranchial 5 originated in the common ancestor of Chondrichthyes + Teleostomi, and was subsequently lost in Osteichthyes (Figure 8).

#### Chondrichthyes

**Figure 9:**
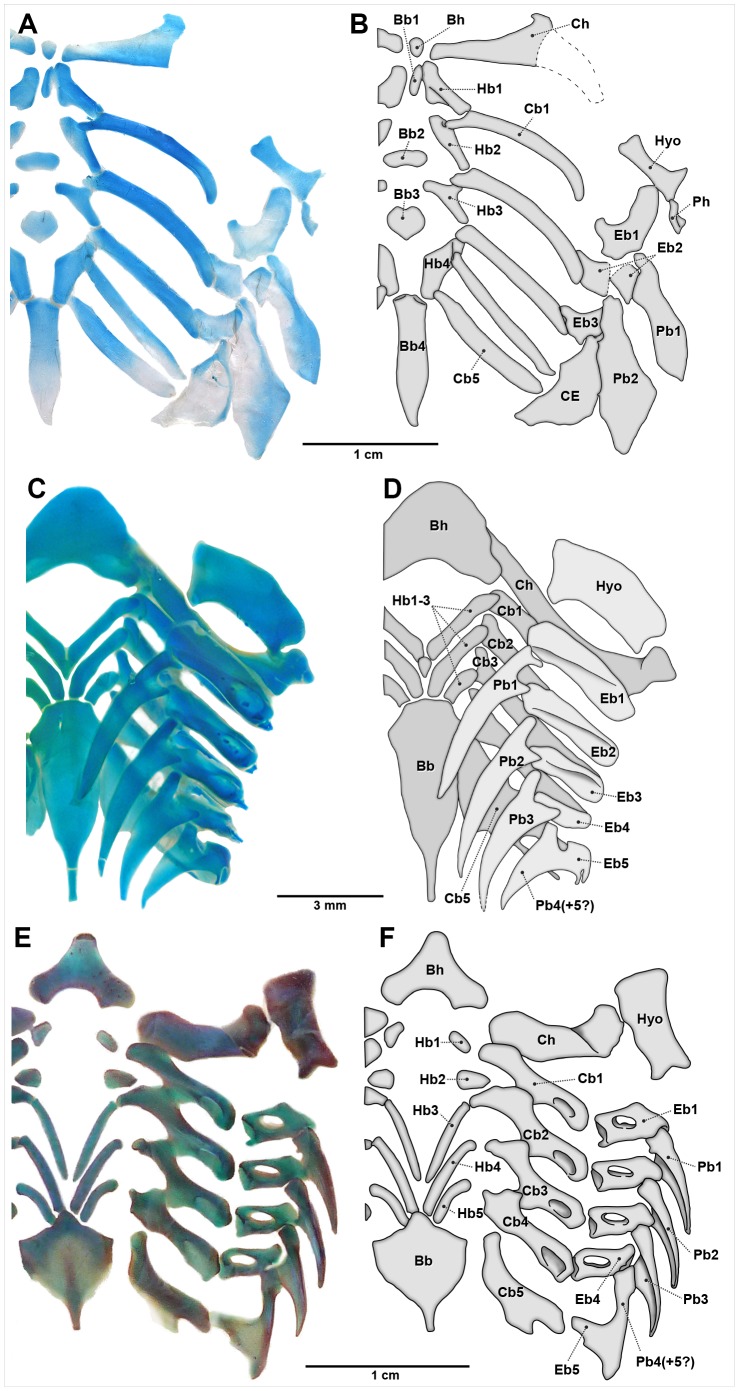
Dorsal view of chondrichthyan branchial arches. Anterior to top. Left-side elements not represented. A) *Callorhinchus capensis* (227 mm SL, ANSP 174852); C) *Rhizoprionodon porosus* (230 mm TL, UFPB 1445.2); E) *Hemiscyllium ocellatum* (213 mm TL, AMNH 38151); B, D, F) respective schematic drawings. Abbreviations: Bb, basibranchial; Bh, basihyal; Cb, ceratobranchial; CE, composite element (Eb4?+Eb5?+Pb4+5); Ch, ceratohyal; Eb, epibranchial; Hb, hypobranchial; Hyo, hyomandibula; Pb, pharyngobranchial; Ph, pharyngohyal.

**Figure 10:**
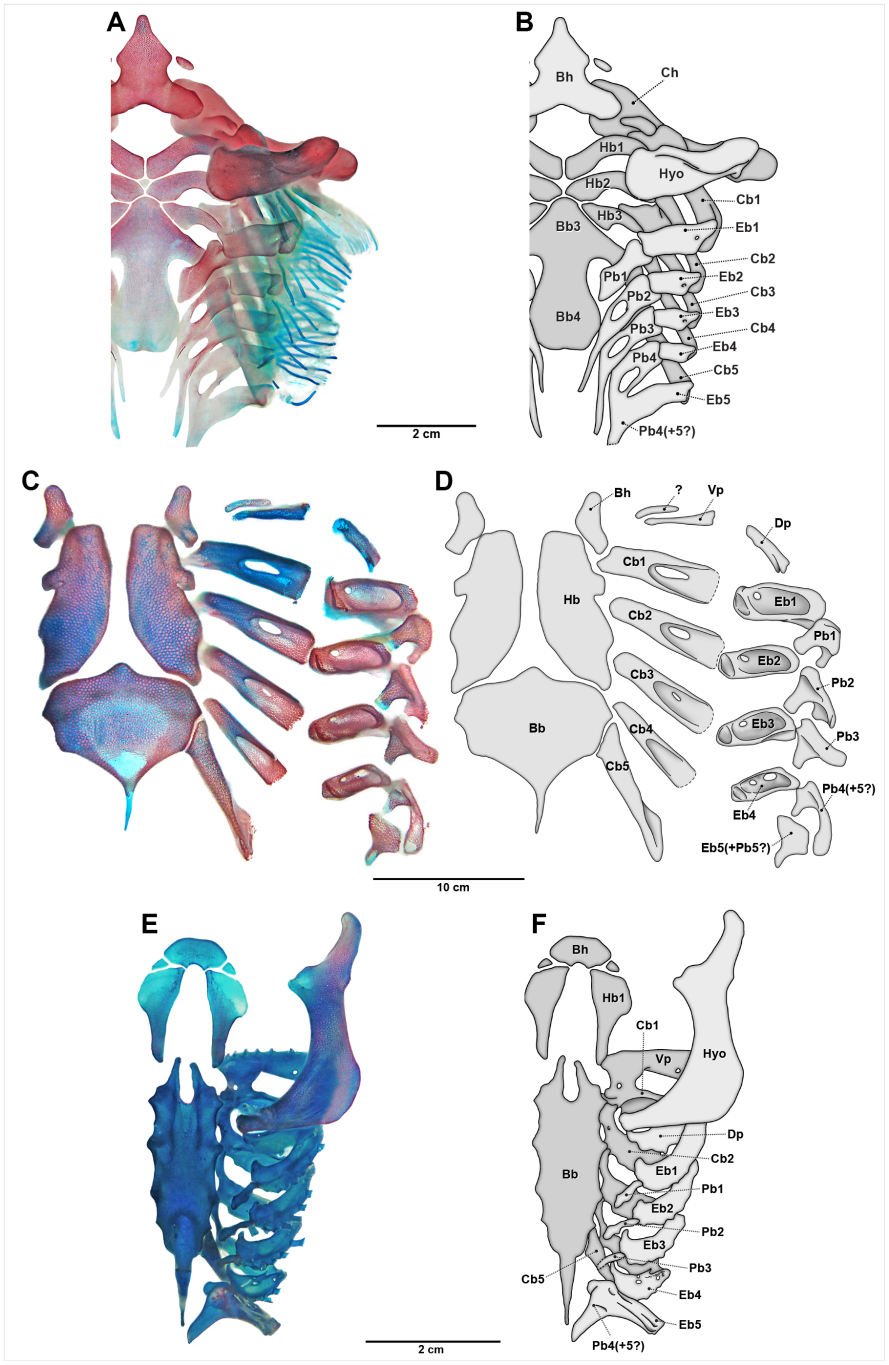
Dorsal view of chondrichthyan branchial arches. Anterior to top. Left-side elements not represented. A) *Squatina californica* (255 mm SL, AMNH 55686); C) *Narcine brasiliensis* (166 mm TL, UERJ 1176.4); E) *Taeniura lymma* (310 mm TL, AMNH 44079); B, D, F) respective schematic drawings. Abbreviations: Bb, basibranchial; Bh, basihyal; Cb, ceratobranchial; Ch, ceratohyal; Dp, dorsal pseudohyoid; Eb, epibranchial; Hb, hypobranchial; Hyo, hyomandibula; Pb, pharyngobranchial; Vp, ventral pseudohyoid.

In extant adult chondrichthyans, epibranchial 5 is usually morphologically distinct from the rod-like anterior epibranchials and is ontogenetically fused with the last one or two pharyngobranchials, forming a composite structure in most taxa [10]–[13], [83] (Figures 9, 10). Fusion patterns of these elements vary among elasmobranch groups, but usually involves pharyngobranchials 4 and 5 [13]. A similar pattern of fusion between the posterior epibranchial and pharyngobranchials is found in chimaeroid fishes [64], [65], [84]. This compound element articulates the branchial basket posteriorly to the pectoral girdle through ligaments (most sharks) or even more directly (batoids, particularly myliobatiforms [12], [13], [85], [86]), and is possibly never an entirely separate element (in those taxa in which epibranchial 5 is not fused with the posteriormost pharyngobranchials, it is tightly articulated to them, e.g. in *Hexanchus* and *Heterodontus* [12], [13]). However, the ventral part of the composite element, corresponding to epibranchial 5, exhibits the same orientation and position as the first four epibranchials. It also has a similar early ontogeny to the preceding epibranchials, being formed entirely by a separate mesenchymal condensation [10], [87], [88]. Even though the patterns of fusion with the pharyngobranchials appear relatively early in ontogeny, the posteriormost dorsal gill arch elements are the last to fully chondrify and are usually less calcified [10], [11], [86]. Irrespective of the above-mentioned variations, the fifth epibranchial is most clearly observed in chondrichthyans among both fossil and living gnathostomes.

The Late Devonian †*Cladoselache* has epibranchials restored as elongate and slender pieces, numbering five or perhaps more [4], [80]. Hybodont sharks, the sister group of neoselachians [35], [89]–[95], also have a separate unmodified epibranchial 5 articulated with ceratobranchial 5 [96]. Epibranchial 5 of the Carboniferous stethacanthid †*Akmonistion* shares the same morphology and relative position of the first four epibranchials, despite being anteroposteriorly broader and not typically rod-like [97]. Coates and Sequeira [97] stated that epibranchial 5 of the Carboniferous ctenacanthiform †*Tristychius* is similar to that of †*Akmonistion*, but its presence is only presumed [98]. Therefore, there is some evidence that epibranchial 5 in stem-chondrichthyans corresponds to the true epibranchial 5, serially homologous to epibranchials 1 to 4, as in living sharks, rays and chimaeroids. No accessory cartilages attached to the distal extremities of the ceratobranchials are known in either fossil or extant chondrichthyans.

#### Osteichthyes

Members of Osteichthyes are divided into two monophyletic groups, the Actinopterygii (ray-finned fishes) and Sarcopterygii (coelacanths, lungfishes and tetrapods) [1], [99].

#### Actinopterygii

The monophyly of this group has not recently been questioned, but diagnostic features vary among authors (see [100] and references therein). In this group, it seems the fifth branchial arch is also primitively incomplete, missing its dorsal parts (epibranchial 5 and pharyngobranchial 5). In fact the presence of epal elements of the fifth branchial arch in the most basal extant and extinct actinopterygian groups, such as Polypteriformes [101]–[103] and †*Cheirolepis*, cannot be ultimately determined. Due to preservation restrictions the accessory element of ceratobranchial 4 in fossil non-neopterygian actinopterygians cannot be verified. Jollie [104] mentioned that in actinopterygians there is generally no epibranchial 5, but he provided no explanation.

**Figure 11:**
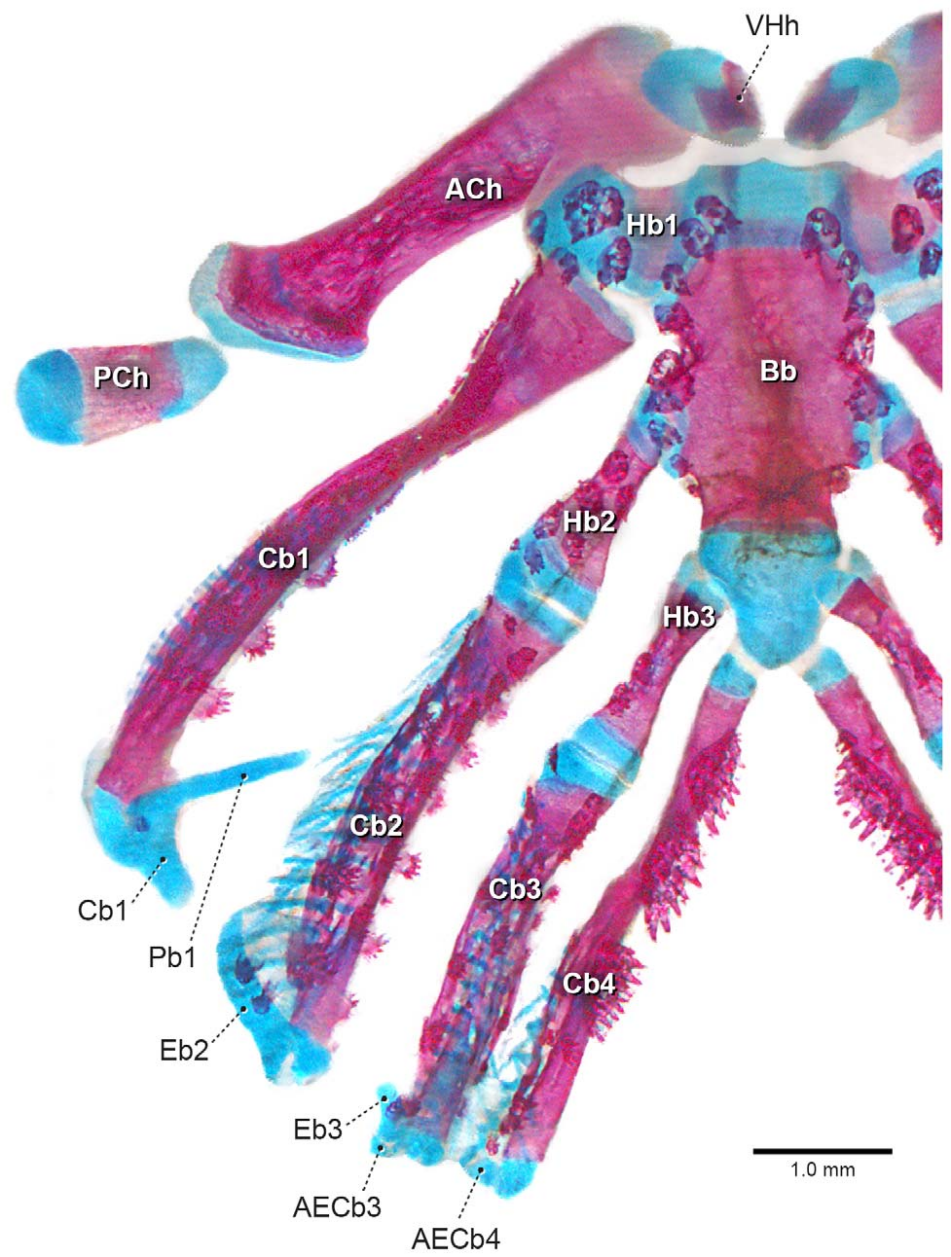
Branchial basket of *Polypterus* sp. (62.5 mm SL, LIRP 7426). Dorsal view, anterior to top. Right-side elements not represented. Abbreviations: ACh, anterior ceratohyal; AECb, accessory elements of ceratobranchials; Bb, basibranchial; Cb1–4, ceratobranchials; Eb1–3, epibranchials; Hb1–3, hypobranchials; Pb1, pharyngobranchial; PCh, posterior ceratohyal; VHh, ventral hypohyal.

Polypteriformes: The peculiar morphology and arrangement of the branchial skeleton of adults of this lineage, considered the most basal extant actinopterygian group [105]–[108] (Figure 8), especially its dorsal elements, have led to the lack of consensus regarding their homologies, resulting in various anatomical terms being applied (e.g. [8], [101], [109]–[112]). The most recent interpretation is that of Springer and Johnson [8]: the two anteriormost dorsal elements, which are rod-like and partially ossified, correspond to pharyngobranchial 1 and epibranchial 1, respectively; the third rod-like and sometimes ossified piece is attached to ceratobranchial 2, being identified as epibranchial 2; the fourth rod-like element is entirely cartilaginous and proximally articulated with the distal cartilaginous cap of ceratobranchial 3, being termed epibranchial 3; epibranchial 4, which is commonly thought to be absent in Polypteriformes (e.g. [101], [109], [113]), is identified as a small cartilage attached to the distal end of ceratobranchial 4; other than the first, no other pharyngobranchial is recognized in *Polypterus*, and no accessory cartilage has been identified. For this group the presence of epibranchial 5 cannot be assessed because its members lack the entire fifth gill arch [114] (Figure 11). This condition could alternatively be interpreted as a terminal step, represented by the loss of the ceratal portion of the fifth gill arch in a transformation series initiated by the loss of the corresponding epal part [101], [102], [109], [115]. Wacker et al. [103] and Springer and Johnson [8] identified a tiny cartilage attached to the distal cartilaginous cap of ceratobranchial 4 as "epibranchial 4", which is commonly thought to be absent in Polypteriformes (e.g. [101], [109], [113]). This piece is here identified as the accessory element of ceratobranchial 4, present in both *Polypterus* and *Erpetoichthys* (Figures 11, 12), due to its position and relation to ceratobranchial 4.

Examination of branchial arches of *Polypterus* and *Erpetoichthys* has led us to raise an alternative hypothesis for the homology of certain elements. We fully agree with Springer and Johnson [8] on the homology of epibranchials 1 through 3, as well as the cartilage articulating with epibranchial 1, also recognized here as pharyngobranchial 1 (Figure 11). However, in addition to the accessory element of ceratobranchial 4, we found separate nodular cartilages at the distal tips of ceratobranchials 3 and 4 also (Figure 12), which we identify as accessory elements. Accessory elements fuse with respective epibranchials in branchial arches 2–3 but remain separate in the fourth arch. This finding explains the peculiar morphology of the second and third branchial arches of Polypteriformes in which epibranchials 2 and 3 form a canal for the passage of efferent branchial arteries in corresponding arches [101], [109]. This condition resembles other actinopterygians in which the accessory cartilaginous element of ceratobranchial 4 is fused with the cartilaginous cap of ceratobranchial 4, forming a canal for the most posterior efferent artery [14], [16], [116]. Although published data on branchial circulation is scant regarding this issue, the condition in *Amia*, in which the four efferent branchial arteries run along the dorsal surface of epibranchials 1–4, extending ventrally along the ventral surface of ceratobranchials 1–4 but without passing through any canal formed in the articular region between dorsal and ventral branchial arch elements [cf. [117], may be the generalized actinopterygian state. Moy-Thomas [115] did not find accessory cartilages of ceratobranchials in his ontogenetic study of *Polypterus*. In addition, the presence of only three epibranchials and a single pharyngobranchial in *Polypterus*, which are totally cartilaginous or weakly ossified, may be attributed to a delay in development. Indeed, Moy-Thomas [115] reported a 9.3 mm larva of *Polypterus* with all four ceratobranchials formed but lacking any vestige of epibranchial elements. The next stage of *Polypterus* available to Moy-Thomas [115] was a 30 mm larva, in which epibranchials 1 and 2 had just started to form and there was still no sign of the third epibranchial. This suggests a putatively heterochronic event in epibranchial formation in *Polypterus*, since the epibrachial of many actinopterygians appear early in ontogeny, just after the formation of all ceratobranchial elements (e.g. [42], [48], [49], [118], current study).

Extinct stem actinopterygians: In the well preserved Late Devonian †*Mimipiscis* and †*Moythomasia* there are only three epibranchials articulated to the first three ceratobranchials [107]. Gardiner [107] indicated that epibranchial 4 of †*Mimipiscis* may have been cartilaginous, as in *Latimeria*. According to [119], only the four anterior epibranchials are present in the well-preserved Triassic †*Pteronisculus stensioi* (treated as †*Glaucolepis stensioi*) (Figure 8). Interestingly enough, Nielsen [119] (fig. 45, pl. 15–17) found in the branchial basket of a single specimen of †*P. stensioi* tubular elements on both sides, which he called "ossifications situated between the visceral arches". The anteriormost element lies behind the distal end of the hyomandibula. The remaining bones are located posterior to the region of articulation between the ceratobranchials and epibranchials 1 to 3. These ossicles are remarkably similar in size, shape and relative location to the accessory element of ceratobranchial 4, and are therefore possibly serially homologous. In branchial arches 4 and 5, such elements are missing, although they may have been present as cartilages not preserved during fossilization. The Triassic paleonisciform †*Birgeria*, which has close affinities with Acipenseriformes [32], [38], has the first four epibranchials [120].

Acipenseriformes: This group is the sister taxon of living non-polypteriform actinopterygians (cf. [121]). A separate accessory element of ceratobranchial 4 is not reported in sturgeons (Acipenseridae) and paddlefishes (Polydontidae) ([8], [109], [122]–[127], current study). However, the illustration in Hilton et al. [125] of the ventral portion of the branchial basket of an 85.5 mm SL specimen of *Acipenser brevirostrum* has called our attention to a possible condition that went unnoticed. At the distal extremity of ceratobranchials 1–4, there is a region that, despite not being detached, has a nodular aspect, especially in the fourth arch. It is also noticeable that there is a ligament connecting this prominence to the distal tip of ceratobranchial 5, in a configuration very similar to that presented by teleosts. In a 23.3 mm SL specimen of *Acipenser brevirostrum*, such projections on the distal ends of ceratobranchials 1–4 are not present ([125], fig. 72A), and exhibit the conventional quadradular shape, indicating that nodular extremities of ceratobranchials 1–4 appeared later in phylogeny. Interestingly, as reported in this study, a similar condition occurs in the ceratobranchial 4 of *Amia*. These structures may be non-detached precursors of the accessory element of ceratobranchials 1–4, although a more reliable conclusion must await a developmental study of the branchial arches of *Acipenser*.

Lepisosteiformes: This order contains the gars, which, with Amiiformes, comprise the Holostei, itself the sister group of Teleostei (*sensu* [33], [128]). Among lepisosteiforms, no epibranchial 5 and accessory element articulated to the distal tip of ceratobranchial 4 are known to occur ([33], [129], [130], current study). However, Springer and Johnson [8] reported for *Atractosteus* paired accessory cartilages attached to the double-headed distal extremities of ceratobranchials 1 and 2. In the revision of the Lepisosteiformes by Grande [33], small but distinct cartilaginous nodules are shown at the distal ends of ceratobranchials 1 to 3, on both sides of the gill basket, in *Lepisosteus osseus* (fig. 64a) and *Atractosteus spatula* (figs. 256a–b).

Amiiformes: Jollie [131], when describing the development of the head of *Amia*, did not mention or illustrate any cartilages articulated with the distal extremities of ceratobranchials. All ceratobranchials of *Amia* are distally cartilaginous, with the cartilaginous cap of ceratobranchial 4 of adults slightly enlarged in relation to other ceratobranchials; ceratobranchial 4 also has a medial expansion (e.g. van Wijhe [109]: pl. 16, fig. 13; Allis [117]: pl. 33, figs. 49–50; Grande and Bemis [132]: fig. 53A), a condition similar to *Acipenser* (see above). However, ceratobranchial 4 of very young individuals of *Amia* has no detectable distal expansion, showing the same aspect of the other ceratal elements (with distally tapered tips; Grande and Bemis [132]: fig. 53K; current study). This indicates that the appearance of the medial expansion of the distal tip of ceratobranchial 4 is a late event in the development of the branchial arches of *Amia*. If the condition in *Amia* is primitive, a transformation series in which the accessory element does not separate from the distal aspect of ceratobranchial 4 and leads to an independent accessory element of ceratobranchial 4 is feasible. Allis [117] reported that sometimes there is a small cartilaginous piece attached to the distal cartilaginous cap of ceratobranchial 5 (identified as epibranchial 5), although this element has not been found in other studies of *Amia* (e.g. [109], [131], [132]) and was not identified in our specimens. This element may represent an accessory cartilage instead of an epibranchial.

**Figure 13:**
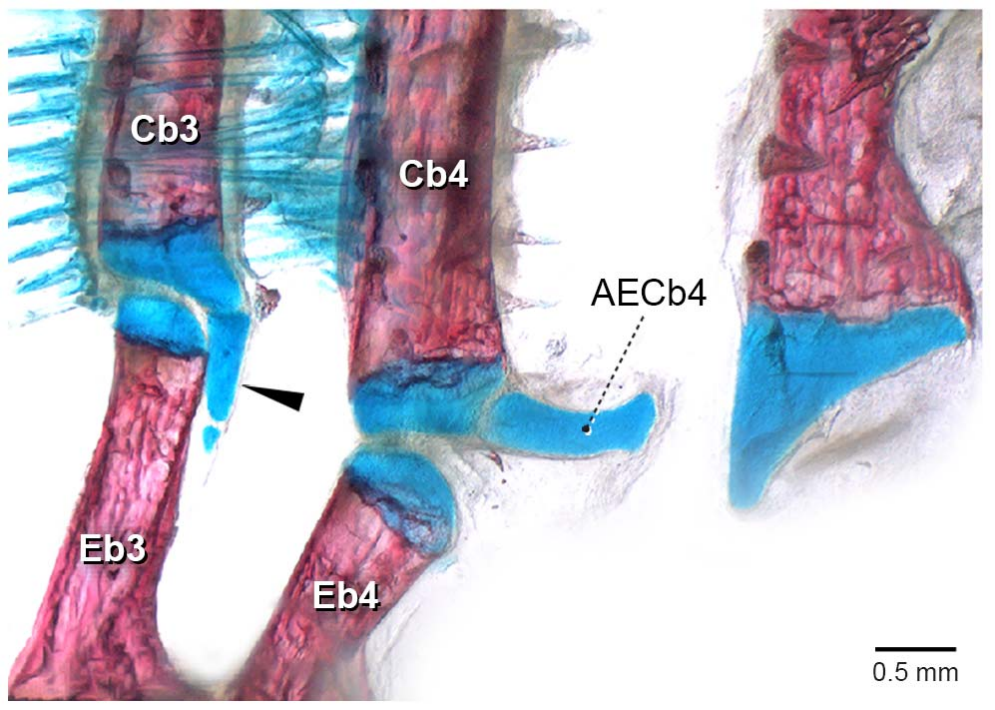
Posterior portion of the last right side branchial arch of *Pimelodus ortmanni* (88.1 mm SL, LIRP 10053). Arrowhead points to the accessory cartilaginous element of ceratobranchial 3. Abbreviations: AECb4, accessory element of ceratobranchial 4; Cb3–5, ceratobranchials; Eb3–4, epibranchials.

Teleostei: Among living teleosts, the accessory element of ceratobranchial 4 is found in several disparate groups, and frequently referred to as epibranchial 5. This is usually a cartilaginous element located at the distal tip of ceratobranchial 4, very close to the articulation with epibranchial 4. Conversely, an element typically connected with the distal tip of ceratobranchial 5 and dorsally oriented, identified as epibranchial 5 (e.g. [23], [27], [133]), is rarely present. In reviewing the homologies of the elements of the last two gill arches that are linked to the epibranchial organs, Pasleau et al. [24] considered the cartilaginous element of ceratobranchial 4 described for gonorynchiforms, clupeiforms and other teleosts as being a polyphyletic neoformation, although they did not offer any objective basis for this assumption.

The posterodorsal part of the gill arch skeleton in representatives of all major teleostean groups was illustrated by Nelson [15]. He indicates that epibranchial 4 and accessory element of ceratobranchial 4 are primitively discrete elements in teleosts. The accessory element of ceratobranchial 4 is always a small oval or cylindrical cartilage articulating with the posteroventral end of epibranchial 4, and epibranchial 4 is a relatively simple bone, anteriorly narrow and with a cartilaginous posterior expansion that more or less follows its contour. Epibranchial 4 assumes a variety of distinctive shapes and connections with the accessory element of ceratobranchial 4, which are characteristic of particular lineages [16]. Often, these distinctive modifications are associated with the development of a particular structure, such as an epibranchial organ for the concentration of small food particles [15], [134], [135].

Among euteleosteomorphs, epibranchial 4 primitively has several features: 1) posteroventrally, above its articulation with ceratobranchial 4, it supports the accessory element of ceratobranchial 4; 2) anteroventrally, it supports the fifth upper pharyngeal tooth plate, which forms the main upper pharyngeal dentition along with pharyngobranchial 4; 3) anteriorly it articulates directly with pharyngobranchial 4; 4) dorsally, it forms an elevation or specific process for insertion of the fourth external *levator* muscle; and 5) anterior to this elevation, on the dorsal edge, it bears a short uncinate process that forms the pylon of a connective tissue bridge between it and a corresponding uncinate process on epibranchial 3. Hence, epibranchial 4 is mechanically connected to epibranchial 3 via the uncinate processes, is joined to the basicranium by the fourth external *levator* muscle, and it supports part of the main upper pharyngeal dentition in primitive euteleosts [16].

Springer and Johnson [8] described the accessory element of ceratobranchial 4 as varying from being completely discrete to partially fused with the distal end of epibranchial 4, or infrequently, with the distal end of ceratobranchial 4. This corroborates Rosen's [16] description of the accessory element of ceratobranchial 4, which primitively (in basal teleosts) collaborates to form a notch through which the fifth efferent artery passes (formed by the fusion of the ventral corner of epibranchial 4 and the accessory element of ceratobranchial 4). Rosen also described patterns for this fusion wherein the accessory element of ceratobranchial 4 can be partially to completely fused with epibranchial 4.

Below we summarize the states of the accessory element of ceratobranchial 4 in major higher-level clades of extant teleosts (taxonomic organization follows [41]):

Osteoglossomorpha: In *Hiodon* (Hiodontiformes), Hilton [136] did not describe any trace of the accessory element of ceratobranchial 4, and we confirm that there is none. The distal cartilage of ceratobranchial 4 is tapered. In its sister group, Osteoglossiformes, the cartilaginous accessory element of ceratobranchial 4 is absent in *Osteoglossum* specimens examined, but present, although faintly stained, in the examined *Arapaima gigas*. It is evident in Hilton's [137] illustrations of the epibranchials of *Pantodon* and, possibly, also present in *Heterotis*, where it is described as a “medially directed element that contacts the fourth branchial arch at the distal point where epibranchial 4 and ceratobranchial 4 meet”. It is also present in *Notopterus* Ridewood ([138], fig. 17).

Elopomorpha: In Elopiformes, the most basal elopomorph clade, we found an inconspicuous accessory element of ceratobranchial 4 in *Megalops* (Megalopidae). *Albula* and *Pterothrissus* are also reported to have a discrete accessory element of ceratobranchial 4 [8]; in *Albula*, this element is fused to ceratobranchial 4 [8, current study]. These genera belong to the order Albuliformes (Albulidae), a basal clade within Elopomorpha (*sensu* [139]). In the more derived Anguilliformes, which comprises roughly 95% of elopomorph taxonomic diversity, Nelson [140] did not mention or illustrate any cartilaginous element at the position of accessory element of ceratobranchial 4, for any anguilliform lineage. Some recent papers dealing with branchial arches for species of this group also do not report this element [141], even in its basalmost family, the recently described Protanguillidae [142]. However, the fifth gill arch of some congroids and anguilloids is subjected to varying degrees of developmental truncation, even reaching a condition of complete loss of ceratobranchial 5 (e.g. [14], [114], [140]).

Otomorpha: According to Wiley and Johnson [41], this group comprises two subdivisions: Clupei and Ostariophysi. The monophyly of this group is supported by both molecular and morphological analyses [41], [143]–[146].

Clupei: The cartilaginous accessory element of ceratobranchial 4 was identified in all specimens examined (*Denticeps*, *Thryssa*, *Hilsa*), and has been reported for certain members of this group (e.g. [19], [147], [148]). Di Dario [149] described this element as present in all specimens he examined. The accessory element of ceratobranchial 4 can be ontogenetically fused with epibranchial 4 (as also reported by [59]) forming distinct patterns (dorsally only or ventrally and dorsally). The complete fusion creates a notch for the passage of the efferent branchial artery, also identified by Nelson [15] in some clupeomorphs.

Ostariophysi: The accessory element of ceratobranchial 4 is also primitively present in this group, and is widely reported in the literature for many ostariophysan groups. In the basal Gonorynchiformes, accessory element of ceratobranchial 4 is found in virtually all genera, and almost always identified as epibranchial 5 (e.g. [23], [27], [42], [150], [151], current study). Monod [27] coined the name “cartilage semi-lunaire” for that piece of the fourth branchial arch of *Gonorynchus*. Thomas [152] figured the branchial arches of *Chanos* and showed the clear relation of accessory element of ceratobranchial 4 with the cerato- and epibranchial 4 articulation. Although she also identified that element as epibranchial 5, it was cited between quotes [152], revealing her doubt about its homology. Britz and Moritz [153] did not report the accessory element of ceratobranchial 4 for the miniature African kneriids *Cromeria nilotica* and *Grasseichthys gabonensis*, but they distinctly illustrated it for *Cromeria occidentalis* as a cartilaginous splint identified as epibranchial 5 (see fig. 10E). Exceptionally, in 17–20 mm SL juveniles of *Chanos*, the accessory cartilage of ceratobranchial 4 starts to ossify perichondrally [42], [43].

Among Otophysi, the accessory element of ceratobranchial 4 is also present and relatively well documented. Engeman et al. [49] described in detail the development of the pharyngeal arch skeleton in *Catostomus* (Cypriniformes), in which the development of the accessory element of ceratobranchial 4 is well documented (confirmed in our examined material). Many other papers have also reported this element in species of this order [154]–[157]. In Characiformes the accessory element of ceratobranchial 4 is also reported (in text and illustrations) in several papers (e.g. [26], [30], [158], [159]). Daget [30],[160] noticed the anatomical differences of the “epibranchial 5” of Characiformes, naming it the epibranchial accessory. Among siluriforms, a nodular cartilage next to the distal tips of cerato- and epibranchial 4 is usually reported (e.g [17], [18], [161]). Recognizing its close association with the ceratobranchial 4, Bockmann and Miquelarena [28] interpreted it in a heptapterid catfish as a neomorphic structure. de Pinna [162] also commented that the accessory element of ceratobranchial 4 is ossified in a large individual of Helogenidae. He also noted that for Siluriformes, the “remnant of epibranchial 5” ( =  accessory element of ceratobranchial 4) is primitively present, as a small cartilaginous nodule, close to the posterior cartilage of ceratobranchial 4. In some taxa it has been secondarily lost. The condition we report for Siluriformes was also confirmed by Britto [163]. Within Gymnotiformes, the accessory element of ceratobranchial 4 is present [164]–[167].

Euteleostomorpha: This group comprises teleost clades that, together, form the sister group to Otomorpha [41], and is divided in two subgroups, Protacanthopterygii (*sensu* [168]) and Neoteleostei.

Protacanthopterygii: This taxon comprises Argentiniformes (comprising Argentinoidei and Alepocephaloidei) and Salmoniformes (including Esocoidei *sensu* [99] and Osmeroidei) [41]. The accessory element of ceratobranchial 4 is present in many protacanthopterygian species, having been described in several papers [8], [16], [20], [21], [169]–[172] and observed by us in *Salmo*. However it was not reported for *Esox* by Jollie [104] nor could we find it in our examined material. As usually happens in other unrelated groups, the accessory element of ceratobranchial 4 may be associated with the last efferent branchial artery, forming a specific notch [16], [173]; therefore, it could be fused to epibranchial 4, encircling the artery. The patterns of these fusions vary, and may be independent for each group (see [21]).

Neoteleostei: The Neoteleostei, *sensu* Wiley and Johnson [41], who moved Esociformes to Protacanthopterygii, was diagnosed by Rosen [174] as a monophyletic group, comprising Stomiiformes, Aulopiformes, Myctophiformes, and Acanthomorpha, in successive phylogenetic order [175]–[177]. Among Stomiatia ( =  Stomiiformes *sensu* [178]) the literature is usually not clear about the presence of the accessory element of ceratobranchial 4 [179], [180]. Nonetheless, in a recent paper by Schnell and Johnson [181] this element is clearly evident in several species within this group. For the Aulopa ( =  Aulopiformes *sensu* [21]), a large cartilaginous accessory element of ceratobranchial 4 is considered primitively present and secondarily reduced or lost in some groups [182].

Ctenosquamata: Ctenosquamates comprise Myctophata ( =  Myctophiformes) and Acanthomorphata ( =  Acanthomorpha) [41], [177], [183], the latter being the crown group and major radiation of extant teleosts (with about 300 families and over 14,000 species, they encompass the majority of living teleosts [184]). The accessory element of ceratobranchial 4 was reported to be absent for all ctenosquamates [182]. Indeed, no cartilaginous structure similar to the accessory element of ceratobranchial 4 was documented by Stiassny [183]. However, the accessory element of ceratobranchial 4 is commonly present in Ctenosquamata, at least in Percomorphacea of Acanthomorphata (*sensu* [41]). Hilton et al. [29] described and illustrated a small cartilaginous piece located posteriorly to the distal end of epibranchial 4 in several genera of the Carangiformes, which the authors called "accessory cartilage", but they did not provide any comment on its homology. It can be seen in the arches, when dorsal and ventral elements are articulated, that the accessory cartilage is closely connected to the distal end of ceratobranchial 4. Mok and Shen [185] represented a “basal cartilage of the fourth ceratobranchial” in representatives of several families of “Perciformes”, which is restricted to the Percoidei *sensu* [186] (see [41]) (e.g. Chaetodontidae, Drepanidae, Kyphosidae, Monodactylidae, Pomacanthidae, Toxotidae), and Acanthuridae (Acanthuriformes). We have found a very conspicuous accessory element on ceratobranchial 4 in *Atherinella brasiliensis* (Atherinidae), a member of Atheriniformes, which is nested within the Smegmamorpharia (*sensu* [41]). The cartilaginous distal caps of ceratobranchials 2–4 of the adrianichthyid *Oryzias* (Beloniformes), also belonging to Atherinomorphae, are greatly expanded ([187]: fig. 14), and could be interpreted as non-segmented accessory elements. A similar condition is also found in the cyprinodontiform *Anableps* (Atherinomorphae) examined. Small, cartilaginous nodules are doubtlessly associated with the terminal portion of ceratobranchial 4 in the sciaenids *Larimus breviceps* and *Paralonchurus brasiliensis* (current study).

There is substantial variation in the shape of the accessory element of ceratobranchial 4, which can be fused with the distal tip of ceratobranchial 4, leading to an elongate cartilaginous posterior aspect or a completely detached and discrete element. Taking into account the morphology, position, and relationships with surrounding elements of the small cartilaginous piece described and illustrated by Mok and Shen [185] and Hilton et al. [29], there is no reason not to recognize it as homologous with the accessory element of ceratobranchial 4. We strongly suspect that when a more complete search of the accessory element of ceratobranchial 4 is undertaken, its presence will be verified in several other families of Percomorphacea.

#### Sarcopterygii

Living fish-like sarcopterygians are the coelacanth *Latimeria* (Actinistia) and lungfishes *Lepidosiren*, *Neoceratodus* and *Protopterus* (Dipnoi). Both *Latimeria* and *Neoceratodus* have four typical epibranchials while their fifth arch lacks an epibranchial [8], [14], [188]–[191]. In *Lepidosiren* the five branchial arches are slender cartilaginous bars ([192–194,195, current study). The branchial bars of *Protopterus* are heavily reduced, lacking all epibranchials other than those of the two first arches, which are also cartilaginous [196]. The reduction of the branchial basket is even more pronounced in *Lepidosiren*, in which there is no epal element [196, current study]. This reduced condition likely resulted from paedomorphosis, which may have played a major role in the evolution of the Dipnoi [197]. No accessory element attached to the distal tip of ceratobranchial 4, or to any ceratobranchial, has been reported and/or illustrated for living coelacanths and dipnoans [188], [189], [193], [194], [198]. Reduction of gill arch elements continued in tetrapods, with the loss of the fifth ceratobranchial and all epal elements in all living amphibians [189], [199], [200].

Stem-group, fish-like sarcopterygians are known from fossils that rarely have their branchial arches preserved, especially the posteriormost arch, a result of either a natural reduction (the loss is expected considering that tetrapods suffer drastic losses of gill elements, including the entire last arch [189], [200]) or poor preservation. Despite being exceptionally well preserved, branchial arches of stem sarcopterygians, such as the Devonian †*Ligulalepis* and †*Meemania* and the Silurian †*Guyiu* and †*Psarolepis* [201]–[204], have not been found. Data on the branchial basket of Actinistia other than the extant genus *Latimeria* is scarce. John Long (pers. comm.) reported that the coelacanths from the Gogo Formation in Australia also have five large arches. Gill arches of the Devonian Onychodontiformes, the most basal lineage within the crown Sarcopterygii, is known from a single species, †*Onychodus jandemarrai*, also from the Gogo Formation, which has four ossified arches (John Long, pers. comm.). Four gill arches were described for the porolepiform †*Glyptolepis* but epibranchials, if present, were not preserved [205], [206], while five were reported for the porolepiform †*Laccognathus* [207]. The closely related Devonian dipnoan †*Griphognathus* was reconstructed as having four gill arches, with four epal elements clearly preserved (no epal components, and only three ceratobranchials, are preserved in †*Chirodipterus* and †*Holodipterus* [208]). The Devonian osteolepiform †*Eusthenopteron*, one of the best morphologically known sarcopterygian fishes [73], [209], has four branchial arches with three epibranchials associated with the three anteriormost ceratobranchials; sometimes its fifth arch is artistically depicted but it is not actually present in the fossils [73], [189], [209]. The branchial basket of the osteolepiform †*Mandageria* appears to be complete and was interpreted as also having four arches; epibranchials were not mentioned [206]. In the Devonian tetrapodomorph †*Gogonasus*, supposedly the sister taxon of Elpistostegalia, the fifth arch is lost while the fourth is atrophied [John Long, pers. comm.]. Details of the branchial baskets of fish-like tetrapods generally treated as elpistostegalians, namely the Late Devonian genera †*Elpistostege*, †*Panderichthys*, and †*Tiktaalik*, are poorly known [210].

The branchial arches of the earliest sarcopterygian tetrapods, such as the Late Devonian †*Acanthostega*, †*Ichthyostega*, and †*Ventastega*, are either unknown or poorly preserved, precluding our understanding of their epal elements, if present [211], [212]. Coates and Clack [211] illustrated only ceratal elements and no epal component in †*Acanthostega*. Clack et al. [213] depicted a specimen of †*Ichthyostega* that appears to have four ceratal gill components; but again, no epal element is noticeable. The Permian temnospondyl †*Dvinosaurus*, which appears to have a completely preserved branchial basket, has only four paired arches lacking epal elements [214]–[216].

The most reliable information about gill arch morphology in basal sarcopterygians is obviously that observed in *Latimeria*, *Lepidosiren*, *Neoceratodus*, and *Protopterus*. Considering that the fossil record of gill arches of sarcopterygians, although poor, does not contradict the data observed in its few living members, it is presumed that the primitive condition for their gill arches is one in which neither a true epibranchial 5 nor an accessory element of ceratobranchial 4 were present (Figure 8). Moreover, the loss of the fifth arch may be a synapomorphy for advanced sarcopterygians, including at least osteolepiforms, tetrapod-like elpistostegalians, and tetrapods (Figure 8). The absence of remaining epal elements (1–4) was considered a synapomorphy for crown tetrapods by Schoch and Witzmann [200], although this loss may be more general, perhaps at the elpistostegalian level.

### Evolution of epibranchial 5 and accessory element of ceratobranchial 4 in gnathostomes: a phylogenetic perspective

The true epibranchial 5, directly attached to ceratobranchial 5, is present in the chondrichthyans and the acanthodian genus †*Acanthodes*, and possibly in the acanthodian †*Halimacanthodes* [34], [82]. Within Osteichthyes, its absence was reported for all living neopterygians plus its sister group, the Acipenseriformes, although the condition in stem actinopterygians cannot be determined due to poor fossilization of their branchial arches and the absence of the fifth branchial arch in polypteriforms (Figure 8). Epibranchial 5 is also lacking in the Sarcopterygii, as is known in its few living fish-like representatives, *Lepidosiren*, *Neoceratodus*, *Protopterus*, and *Latimeria* [8], [188], [189], [192]–[194], [196], [198]. As a consequence, despite the lack of data on epal elements of successively more basal actinopterygians (fossil stem-groups), the loss of epibranchial 5 may be confidently interpreted as a synapomorphy of Osteichthyes (Figure 8). In fish-like sarcopterygians reductions in the branchial basket have also taken place, resulting in the total loss of the posterior arches (e.g. [189], [217]). One can speculate that this may be the result of decreased pharyngeal ventilation as basal sarcopterygians moved toward shallow, deoxygenated waters [218].

The presence of an accessory element, normally cartilaginous, attached to the distal extremity of ceratobranchial 4, is interpreted as a synapomorphy for Teleostei (Figure 8), and the occurrence of a very small cartilage in the fourth gill arch in Polypteriformes [8], [103] is considered homoplastic (Figure 8).

As mentioned above, anatomical evidence from the adult branchial skeleton and muscles indicate that the so-called epibranchial 5 in Teleostei is in fact a novel structure, being therefore renamed accessory element of ceratobranchial 4 (Figure 8). This hypothesis is reinforced by two findings from our developmental studies: the relatively late development of that element when compared to the early ontogenetic emergence of the epibranchial series, and its origin from a chondroblastic layer shared with ceratobranchial 4, leading to the conclusion that it is formed by segmentation of the distal cartilaginous tip of ceratobranchial 4. Therefore, the “epibranchial 5” in actinopterygians is not homologous to the chondrichthyan epibranchial 5, nor serially homologous to the actinopterygian epibranchial series.

Living members of Acipenseriformes and Amiiformes possess an elongate and medially expanded cartilage at the tip of ceratobranchial 4 that could be the precursor of the accessory element of ceratobranchial 4. The condition in *Amia* and *Acipenser* may represent an intermediate stage towards a separate accessory element. In this case, the presence of accessory element of ceratobranchial 4 would be interpreted as a synapomorphy for Actinopterygii. However, in the absence of ontogenetic evidence, we conservatively do not assume homology between the expanded cartilaginous terminus of ceratobranchial 4 of *Acipenser* and *Amia* and the accessory element of that bone in other actinopterygians.

The presence of accessory elements in the most anterior arches of at least three basal actinopterygian lineages, namely Polypteriformes, †*Pteronisculus*, and Lepisosteiformes (cf. [32], [219]), and possibly in Acipenseriformes, is suggestive that the ancestral *Bauplan* of the visceral arches of the earliest actinopterygians is one in which the serially homologous elements have been repeated in all arches (cf. [220]). In this scenario, the appearance of the accessory elements associated with the four branchial arches should be interpreted as a synapomorphy for Actinopterygii, implying that the accessory element of ceratobranchial 4 in Polypteriformes and Teleostei is homoplastic. Furthermore, loss of accessory elements of ceratobranchials 1–3 would be a synapomorphy for Teleostei, which usually retain only the fourth ceratobranchial accessory element. The absence of accessory elements associated with ceratobranchials, and specifically that of ceratobranchial 4, may be regarded as a result of a heterochronic event due to developmental truncation. However, these conclusions should be taken with caution until a detailed investigation of the anatomy and ontogeny of basal actinopterygians is performed.

Accessory cartilages associated with ceratobranchials other than the fourth may appear also in more advanced actinopterygians, as in teleosteans (for a survey of these elements see Table 8 in [8]), but probably as independent reversals to primitive states. Due to their similar morphologies and attachments to other ceratobranchials, we believe these elements are serially homologous to the accessory element of ceratobranchial 4. We have consistently found in some catfishes of the family Pimelodidae (e.g. *Pimelodus*) an independent cartilage attached to the cartilaginous cap of ceratobranchial 3 at its posteromesial border, and aligned to epibranchial 3 (Figure 13). An accessory cartilage associated with ceratobranchial 5 was also reported in some non-closely related taxa, such as in the clupeomorph *Denticeps* (e.g. [19]), *Gonorynchus* (e.g. [27], [133]) and in alepocephaloids (e.g. [15]). In the case of alepocephaloids, this cartilage forms part of the supporting skeleton for the crumenal organ [15], [20]. Nelson [15] mentioned that this element, called accessory cartilage of ceratobranchial 5 by Greenwood and Rosen [20], may have arisen from the posterior articular surface of ceratobranchial 5, identical to the element here called accessory element of ceratobranchial 4. The presence of these elements is also suggestive of the non-homology of accessory element of ceratobranchial 4 and epibranchial 5, as they demonstrate that an accessory ceratobranchial element may occur simultaneously with an epibranchial in the same arch.

Interestingly, accessory elements of ceratobranchials 1 to 3 of the well-preserved Triassic “palaeonisciform” †*Pteronisculus stensioi* is filled with white calcite, which the author interpreted as evidence that they had undergone perichondral ossification [119]. As mentioned above, the accessory cartilage of ceratobranchial 4 of the gonorynchiform *Chanos* may ossify perichondrally [42], [43]. If so, it differs from the typical endochondral ossification of the elements of the epibranchial series, which can be understood as additional evidence for the non-homology between accessory elements and epibranchials. Nielsen [119] suggested that these ossifications situated between the visceral arches of †*Pteronisculus* could have connected each arch with the arch immediately following. This led him to propose these elements as being serially homologous with the symplectic, which primitively links the mandibular arch with the hyoid bar. An investigation of the homology of the accessory elements of ceratobranchials with the intervening pieces of the mandibular and hyoid arches (symplectic and interhyal, respectively), is beyond the scope of this article, but is being prepared for publication elsewhere.

## Conclusions

In our study comparative morphology of adults and ontogenetic data did not support the homology between epibranchial 5 and accessory element of ceratobranchial 4. These situations, in which a structure of a given taxon (accessory element of ceratobranchial 4 in teleosts) is roughly similar to a non-homologous structure of another taxon, while the original element is actually absent (epibranchial 5), may lead to false homology statements (i.e. accessory element of ceratobranchial 4  =  epibranchial 5) and, consequently, to an artificial support for grouping. This undesirable situation can be prevented through detailed morphological analysis, including the examination of ontogeny whenever possible, followed by an evaluation of character evolution within a well-supported phylogenetic scheme.
